# The senolytic ABT-263 improves cognitive functions in middle-aged male, but not female, atherosclerotic LDLr^−/−^;hApoB_100_^+/+^ mice

**DOI:** 10.1007/s11357-025-01563-3

**Published:** 2025-02-21

**Authors:** Mélanie Lambert, Géraldine Miquel, Louis Villeneuve, Nathalie Thorin-Trescases, Eric Thorin

**Affiliations:** 1 Faculty of Medicine, Department of Pharmacology and Physiology, University of Montreal, Montreal, Quebec Canada; 2 Montreal Heart Institute, Research Center, 5000 Rue Belanger, Montreal, Quebec H1T 1C8 Canada; 3 Faculty of Medicine, Department of Surgery, University of Montreal, Montreal, Quebec Canada

**Keywords:** Vascular cognitive impairment, Cerebrovascular function, Cellular senescence, SASP, *Inflammaging*, Endothelial function, Morris water maze

## Abstract

Accumulation of cerebral senescent cells may compromise the continuum between vascular and neuronal function, leading to damage and cognitive decline. Elimination of senescent cells might therefore preserve vascular and neuronal functions. To test this hypothesis, we used male and female atherosclerotic LDLr^−/−^;hApoB_100_^+/+^ mice (ATX-mice), a model of vascular cognitive impairment (VCI), treated with the senolytic ABT-263 for 3 months (3- to 6-month or 9- to 12-month old). In young male ATX mice, prevention with ABT-263 improved spatial retention memory, in association with a higher endothelial sensitivity to shear stress and a higher hippocampal CD31^+^ endothelial cell density, lower activation of both astrocytes and glial cells. In young females, ABT-263 tended to improve delayed memory; however, atherosclerotic plaque was magnified by ABT-263, endothelial function was unaffected, hippocampal astrocyte activation increased and expression of CD31^+^ cells decreased. Hence, unlike in males, ABT-263 appears deleterious in young ATX females. In middle-aged males, the curative treatment improved the learning process and memory. Although no change in endothelial function was observed, the benefits of ABT-263 were associated with a decreased expression of several *inflammaging* markers, a higher density of CD31^+^ cells and a lower activation of glial cells. In middle-aged females, ABT-263 induced a surge of *inflammaging* markers, associated with a slower learning process. Altogether, our data demonstrate that ABT-263 differentially affects VCI, improving cognition in male while being deleterious in female ATX mice. More studies are needed to understand the mechanisms at the basis of the sexual dimorphic effects of the senolytic ABT-263.

**Supplementary Information:**

The online version contains supplementary material available at 10.1007/s11357-025-01563-3.

## Introduction

Cognition declines with age and a vascular contribution to neuronal damage, cognitive impairment and dementia has been proposed [1–4]. The brain has high metabolic needs, and proper vascular endothelial function is necessary to maintain the continuum between vascular and neuronal functions to match metabolic demands. Not surprisingly, endothelial dysfunction is a hallmark of vascular aging, characterized by a reduction in flow-mediated dilation (FMD), a reduced capacity to prevent adhesion and aggregation of platelets, and a decreased barrier function [2]. Importantly, endothelial dysfunction is prodromal to cardiovascular diseases [5, 6], including to cerebrovascular diseases [7].

A possible mechanistic link between endothelial dysfunction and cerebrovascular diseases is the age-associated accumulation of senescent endothelial cells (EC). Unlike programmed cellular senescence occurring during embryonic development [8] and transitory by function, chronic stress-dependent cellular senescence is considered as an important contributor to age-related dysfunctions and diseases [9, 10], including in the aging brain [11, 12]. Through the senescence-associated secretory phenotype (SASP), senescent cells promote a chronic, low-grade inflammation [13]. In the context of aging or neurodegenerative diseases, senescence of various cell types in the brain has been reported in astrocytes [14–20], microglia [14, 18, 21–24] and neurons [23, 25–27]. The role of senescent EC in the aging brain is less studied [28–32]. Pharmacological targeting of senescent cells using senolytics, and genetically-driven clearance of senescent cells of all types, increased median lifespan, and preserved organ functions [9], including vascular endothelial function [33, 34] and cognition [14, 25, 35–38]. Hence, accumulation of cerebral senescent cells, including EC, may compromise the continuum between vascular and neuronal function, leading to neuronal damage and cognitive decline.

To test this hypothesis, we used severely dyslipidemic and atherosclerotic LDLr^−/−^;hApoB_100_^+/+^ mice (ATX mice), a model of vascular cognitive impairment (VCI) with established premature cerebrovascular endothelial dysfunction, carotid stiffening, cerebral hypoperfusion and cognitive impairment [39–42]. Differences in cognitive functions between males and females mice were reported [35, 43, 44] or not [45–47]. The sex of the animal model is mostly not considered when investigating the impact of senescence on cognition, but recent studies showed that senolytic treatment only rescued memory in aged male mice [35], in male mice exposed to traumatic brain injury [23], in male rats treated with doxorubicine [48], or showed that senolytic treatment prevented cognitive decline in aged male rats [38], but failed to do so in aged female rats [49]. We therefore tested the effect of the senolytic ABT-263 (Navitoclax) [50, 51] in male and female ATX mice. Since the optimal timing for senolysis is unknown [52, 53], we used two different approaches: 1) an early preventive treatment in young ATX mice, from 3–6 months of age, an age where cognitive dysfunction is mild and possibly reversible [41]; 2) a late curative treatment in middle-aged mice with established and severe cognitive defects [1], from 9–12 months of age. Our goals were to demonstrate that ABT-263 preserves vascular and neuronal functions in ATX mice, validating the causal contribution of senescence to cognitive decline, and test whether this therapeutic concept stands in both male and female mice.

## Methods

### Animals

Male and female dyslipidemic LDLr^−/−^;ApoB_100_^+/+^mice (ATX mice) from our colony [39, 40] were randomly treated from 3- to 6-month of age (mo; preventive treatment) or from 9- to 12-mo (curative treatment), with ABT-263 at 50 mg/kg (Navitoclax; Med Chem Express) or placebo (Phosal 50PG, Lipoid – 60%; PEG400, Sigma-Aldrich – 30%; EtOH—10%). ABT-263 or placebo was administrated by repeated cycles of gavages, for 5 days followed by 2 weeks of rest; during the 3 months of treatment, 4 cycles of gavages were administrated. Mice were sacrificed at 6- or 12-mo, after the preventive or curative treatment, respectively.

All experiments were performed in accordance with the *Guide for the Care and Use of Experimental Animals of the Canadian Council on Animal Care* and the *Guide for the Care and Use of Laboratory Animals* of the US National Institutes of Health (NIH Publication No. 85–23, revised 1996) and the study was approved by the Montreal Heart Institute Ethics Committee (ET No 2019–6203). Mice were kept under standard conditions (24 °C; 12 h:12 h light/dark cycle) and were fed ad libitum with regular chow (2019S; Harlan Laboratories).

### Blood biochemistry

To determine whether chronic treatment with ABT-263 induces any toxicity, blood markers of liver function (glutamic pyruvic transaminase (CPT/ALT-P III), glutamic-oxaloacetic transaminase (GOT/AST-P-III) and alkaline phosphatase) and renal function (plasma creatinine and urea) were measured in mouse plasma. Lipids (total cholesterol, LDL-cholesterol), triglycerides (TG) and glucose levels were also assessed. All analyses were performed in the laboratory of clinical biochemistry at the Montreal Heart Institute.

### Neurobehavioral tests

**Fig. 1:**
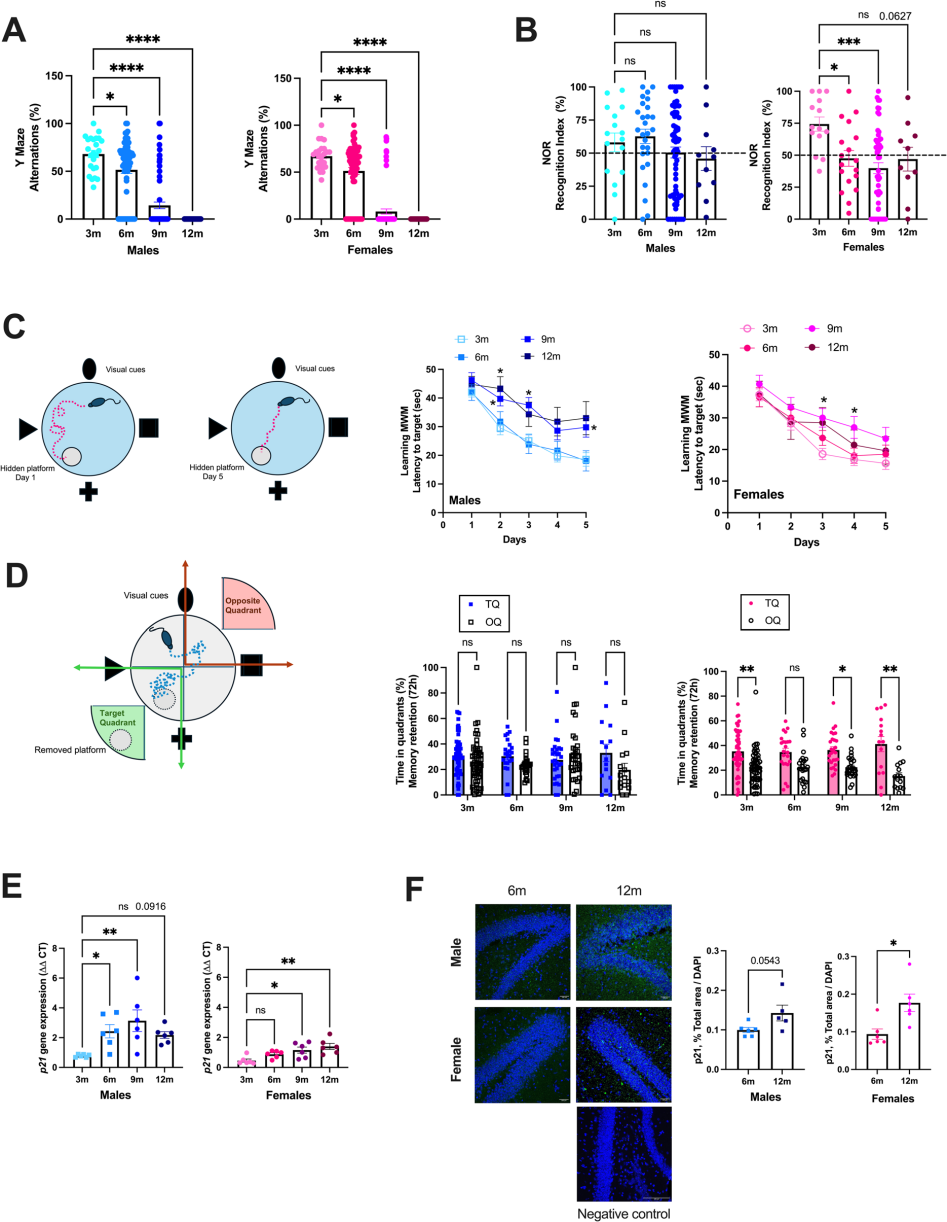
Learning impairment and increased p21 expression with atherosclerosis progression in ATX mice. (**A**) Spatial memory was assessed with the Y maze test in naïve 3 m (*n* = 24 males and *n* = 24 females), 6 m (*n* = 68 males and *n* = 64 females), 9 m (n = 70 males and n = 73 females) and 12 m (*n* = 18 males and *n* = 21 females) ATX mice. *: *p* < 0.05 *vs.* 3 m, one way-ANOVA and Dunnett’s multiple comparisons test. (**B**) Recognition memory was assessed with the novel object recognition (NOR) in naïve 3 m (*n* = 16 males and *n* = 14 females), 6 m (*n* = 27 males and n = 18 females), 9 m (*n* = 64 males and n = 54 females) and 12 m (*n* = 11 males and n = 10 females) ATX mice. *: *p* < 0.05 *vs.* 3 m, one way-ANOVA and Dunnett’s multiple comparisons test. (**C**) Learning memory was assessed with the Morris Water Maze (MWM) test during the acquisition phase over 5 consecutive days, in naïve 3 m (*n* = 55 males and *n* = 56 females), 6 m (*n* = 24 males and n = 24 females), 9 m (n = 30 males and n = 30 females) and 12 m (*n* = 15 males and *n* = 15 females) ATX mice. *: *p* < 0.05 *vs*. 3 m, Two-way ANOVA with repeated measures (Days X Age) and Dunnett’s multiple comparisons test. (**D**) Delayed memory retention assessed during the probe test 72 h after the removal of the platform in the MWM, in naïve 3 m (n = 55 males and n = 56 females), 6 m (*n* = 24 males and *n* = 24 females), 9 m (*n* = 30 males and n = 30 females) and 12 m (*n* = 15 males and *n* = 15 females) ATX mice. *: *p* < 0.05 *vs*. TQ, Two-way ANOVA with repeated measures (Quadrant x Age) and Sidak’s multiple comparisons test. Data are mean ± SEM of n mice. *: *p* < 0.05; **: *p* < 0.01; ***: *p* < 0.001; ****: *p* < 0.0001. (**E**) Gene expression level of the cerebrovascular senescence marker *p21* was quantified by RT-qPCR in 3 m, 6 m, 9 m and 12 m male and female ATX mice (*n* = 6 mice per group). *: *p* < 0.05 *vs.* 3 m, one way-ANOVA and Dunnett’s multiple comparisons test. (**F**) Immunofluorescence of p21 in 6 m and 12 m ATX mice, in males and females (*n* = 6 images *per* age. Representative confocal images (20X), in addition to a negative control (40X), are shown; scale bar = 100 µm, for all images. *: *p* < 0.05 *vs.* 6 m, unpaired t-test)

**Fig. 2:**
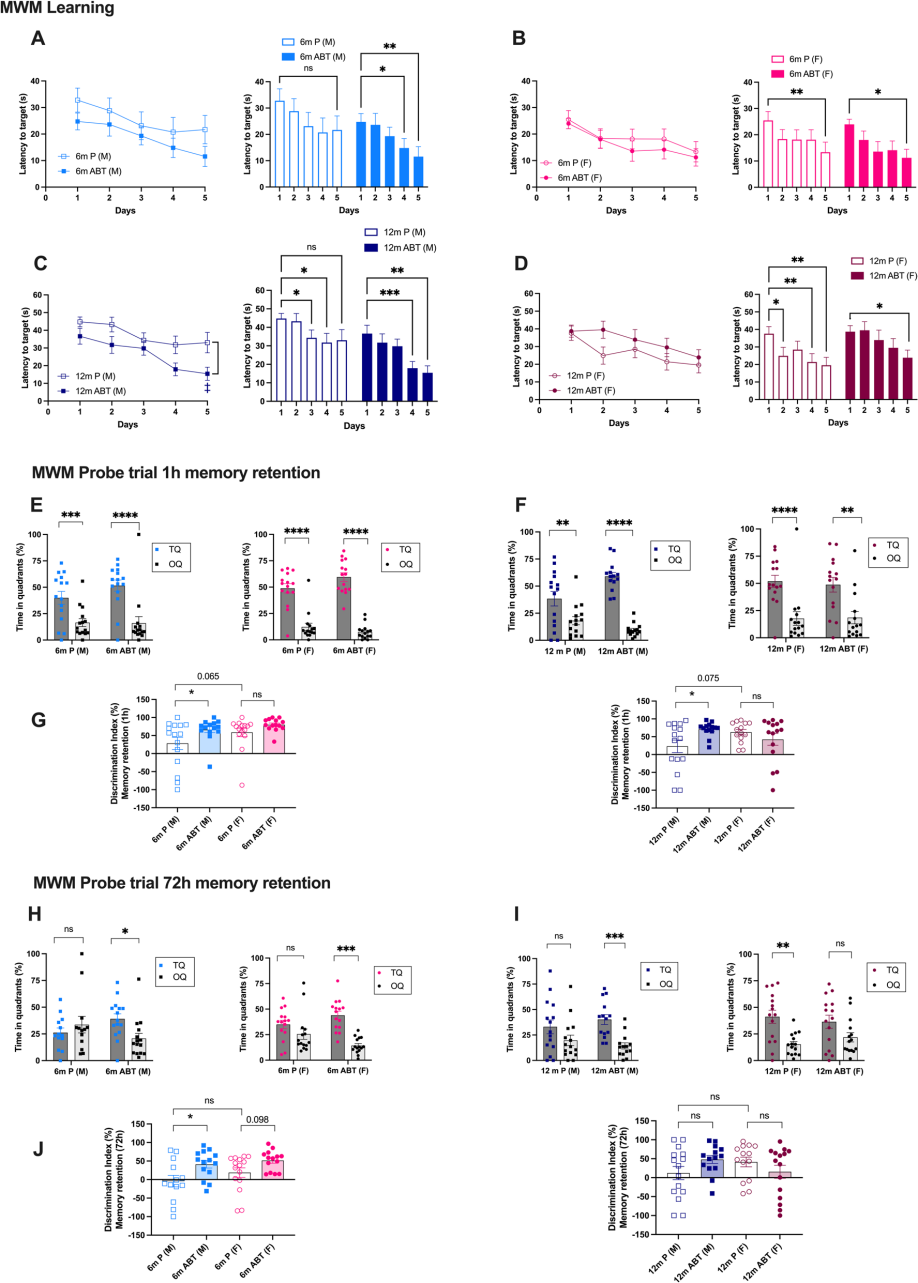
Impact of ABT-263 treatment on cognition in the MWM (**A**) Latency to find the hidden platform from day 1 to day 5 in the acquisition phase (learning memory) of the Morris water maze (MWM) test of male (M, **A**) and female (F, **B**) mice after treatment with placebo (P) or ABT-263 (ABT) at 6-month-old (6 m, **A** and **B**) and 12-month-old (12 m, **C** and **D**). Each value is a mean of 4 trials performed by each mouse, per day of learning. ‡: *p* < 0.05 *vs.* 12 m P (M), Two-way ANOVA with repeated measures (Days x Treatment) and Sidak’s multiple comparisons test. Latency to target was also analyzed to determine in each group at what day the mice started to remember the localization of the hidden platform: * *p* < 0.05 *vs*. Day1, Two-way ANOVA with repeated measures (Days x Treatment) and Dunnet’s test. (**E, F, G**) Percentage of time spent in the target quadrant (TQ, quadrant in which the platform was hidden) and the opposite quadrant (OQ) by male (M) and female (F) mice after treatment with placebo (P) or ABT-263 (ABT) at 6-month-old (6 m, **E**) or 12-month-old (12 m, **F**) during the probe test. This probe test was performed on day 5, 1 h after the last acquisition of the learning phase (1 h memory retention, **E, F, G**) and 72 h after the end of the learning phase (delayed memory retention, **H, I, J**). *: p < 0.05 *vs.* TQ; Two-way ANOVA with repeated measures (Quadrant x Treatment) and Sidak’s multiple comparisons. Discrimination index (%) reflecting 1 h memory retention (**G**) and 72 h memory retention (**J**) in all groups of mice. * *p* < 0.05, Two-way ANOVA (Sex x Treatment) and Sidak’s multiple comparisons test. N = 15 in each group (placebo and ABT-263, males and females). Data are expressed as mean ± SEM of n mice. *: *p* < 0.05; **: *p* < 0.01; ***: *p* < 0.001; ****: *p* < 0.0001

Naïve mice were used to study the effect of age on cognition (Fig. 1). In the subsequent studies comparing the effects of placebo *vs*. ABT-263 on cognition (Fig. 2), mice were evaluated before and then after 3 months of treatment, with either placebo or ABT-263.

*Y-maze test*. The Y-maze test, based on spontaneous alternation behaviour and used to measure spatial working memory and exploratory behaviour, was performed as we previously described [1, 54]. The maze consists of three arms (41 cm long, 16 cm high, and 9 cm wide, labeled A, B, or C) diverging at a 120° angle from the central point. The experiments were performed in a dimly illuminated room, and the floor of the maze was cleaned with super hypochlorous water-soaked paper after each mouse to avoid olfactory cues. Each mouse was placed at the end of the starting arm and allowed to move freely through the maze during a 5-min session. The sequence of arm entries was recorded by means of a computer-based video-tracking system (Smart version 3.0, Panlab/Harvard Apparatus); a mouse was considered to have entered an arm when all four paws were positioned in the arm runway. An actual alternation was defined as entries into the three arms on consecutive occasions. The maximum alternation was subsequently calculated by measuring the total number of arm entries minus 2 and the percentage of alternations was calculated as ((actual alternations/maximum alternations)*100) [1, 54]. Total number of arms entered during the sessions (reflecting locomotor activity) was also recorded. Mice that entered arms less than eight times during the test were eliminated because the data obtained from those mice were not considered to be representative. Experimenters were blinded to groups during data acquisition.

*Novel object recognition (NOR) test*. NOR test is used to assess spatial and recognition memory. The conditions for the test were first validated in untreated, male, 6-month-old ATX mice and then applied for every group of mice. The test comprises 3 trials, performed within the same day in a dimly illuminated room: the habituation trial is an open field in a 50 × 50 cm box and lasts 5 min; the training trial consists of the exploration of 2 identical objects for 10 min; the test trial assesses the exploratory behaviour after the introduction of a novel object, and lasts 10 min. The time separating the habituation to the training trial is 3 min and the time between the training and the test is 6 min. The computer-based video-tracking system Smart (version 3.0, Panlab/Harvard Apparatus) recorded the movement (times [s] spent in exploring familiar *versus* novel object, the total object exploratory times [Σ time on both objects, s], the total distance traveled (cm), and the total mean velocity of movement [cm/s]) of the mice during the 3 trials. A set of boxes and objects were dedicated to female mice and another one to male mice.

For the test be considered functional, 3 criteria must be fulfilled: 1) the time spent on both objects during the training trial should be at least 20 s, as the ability for identifying the novel object will require the memory acquired during exploration of the 2 objects during training, and such aptitude needs exploration of identical objects for significant periods (> 20 s) [55]; 2) during the training trial, the time spent on both objects should be similar; 3) during the test trial, the time spent on the novel object should be significantly higher, demonstrating that mice discriminate the novel from the familiar object. Mice were excluded if they did not move during the test trial, even if they performed adequately the other trials. For each mouse that successfully performed the test, the recognition index (RI, %) was calculated:

$$\text{RI}=(\text{Time spent on the novel object}/\sum \text{time on both objects})*100$$

Experimenters were blinded to groups during data acquisition.

*Morris water maze (MWM) test*. MWM assesses learning and spatial memory [1, 41, 56]. The water-maze apparatus consists of a white circular pool of 150 cm in diameter and 60 cm in height, filled with water made opaque with non-toxic white paint kept at a temperature of 22 °C. A plastic transparent platform (10 cm in diameter) was placed 1.5 cm below the water surface and 40 cm from the edge of the pool, except on day one (habituation phase) where the platform was visible and placed 0.5 cm above the water surface. The entire procedure took eleven days.

Mice were individually transferred from the home-cage to the pool. Release points were balanced across 4 symmetrical positions on the pool perimeter. Each day of the test, mice underwent 4 trials during which they were allowed to freely swim for 60 s or until they found and climbed onto the platform; each trial was spaced from the other by a 30 min inter-trial interval. Platform finding was defined as staying on the platform for at least 3 s.

On day one, during the habituation phase, mice that did not find the platform were trained in locating it by gently placing them on the platform for 30 s at the end of the trial. Then, 48 h later, the acquisition phase started and latency time to reach the platform was measured (repeated for 5 days in a row); mice that did not find the platform were trained in locating it by placing them on the platform for 30 s at the end of the trial.

On the fifth day of the acquisition phase, 1 h and 72 h after the last acquisition trial, the platform was removed from the pool and each mouse was tested for memory retention in a 60-s probe trial. During the probe trials the time spent in the target quadrant (TQ, where the platform was located) *versus* the opposite quadrant (OQ) of the maze was scored as a reliable measure of memory retention 1 h and 72 h after the last acquisition trial. A discrimination index (DI, %) for short (1 h) and delayed (72 h) probe tests was calculated according to:

$$\text{DI}\hspace{0.17em}=\hspace{0.17em}[(\text{Time in TQ}-\text{Time in OQ})/(\text{Time in TQ}\hspace{0.17em}+\hspace{0.17em}\text{Time in OQ})]*100$$

The swim path of the mice and the time spent in the target quadrant were recorded by means of a computer-based video-tracking system (Smart version 3.0, Panlab/Harvard Apparatus). All recordings were automatically quantified, without human intervention. Experimenters were blinded to groups during data acquisition.

### Atherosclerotic lesion quantification

Freshly isolated thoracic aortas of ATX mice were longitudinally opened. With the endothelium facing up, they were fixed with 4% paraformaldehyde (PFA) at 4 °C for at least 24 h. They were then incubated for 1 h with 0.7% oil red O (Sigma-Aldrich # O0625), rinsed 30 s with methanol, incubated 5 min with 0.05% Fast green (Sigma-Aldrich # F7258), and rinsed 2 min with distilled water before pictures were taken. Atherosclerosis lesions were quantified by measuring the red spots in the thoracic aorta using ImageJ software. Plaque areas were expressed as percentage of total aortic area. Experimenters were blinded to groups during data acquisition.

### Cerebrovascular endothelium-dependent dilatory function

Mice were euthanized by terminal anesthesia (3.5% isoflurane in O_2_) followed by exsanguination. Then, mice were decapitated and brains rapidly removed and placed in ice-cold physiological salt solution (PSS; mmol/L: 130 NaCl; 4.7 KCl; 1.18 KH_2_ PO_4_; 1.17 MgSO_4_; 14.9 NaHCO_3_; 1.6 CaCl_2_; 0.023 EDTA; 10 glucose; pH 7.4) aerated with 12% O_2_; 5% CO_2_; and 83% N_2_ at 37 °C. The middle cerebral artery (inner diameter of 100–150 µm, in 6-mo male (125 ± 3 µm) or female (122 ± 3 µm) mice, and in 12-mo male (115 ± 3 µm) or female (123 ± 3 µm) mice) was isolated, transferred to the arteriograph chamber (Living System Instrumentation), cannulated and pressurized at 60 mm Hg for endothelial function assessment as previously described [57]. The artery segment was equilibrated for 30–45 min, and then sub-maximally pre-constricted with phenylephrine (1–3 µmol/L); then dilatory responses were tested with a single cumulative exposure of incremental ramp of shear-stresses (0–20 dyn/cm^2^), by increasing flow rate while maintaining intraluminal pressure constant [57]. Shear stress was applied using the following equation: τ = 4ηQ/πr^3^, where τ is the shear stress (dyn/cm^2^), η the viscosity (0.009 P), Q the flow rate (ml/s) through the lumen, and r the inside radius (cm) [41, 57]. For each imposed shear stress (2, 4, 6, 8, 10, 12, 15, 20 dyn/cm^2^), flow-mediated dilation was quantified by measuring the inner diameter reached at a given shear stress. Then, a maximal constriction, using depolarizing PSS with 127 mM KCl was induced and the minimal diameter measured. Finally, a maximal dilation, using a PSS without CaCl_2_, was induced and the maximal diameter measured. The flow-mediated dilation (FMD) was then normalized, for each arterial segment, to its minimal and maximal diameter and expressed as % of maximal diameter according to the following formula [41, 57]:

$$\text{FMD }({\%})=[(\text{Diameter at a given shear stress }-\text{ Minimal diameter})/(\text{Maximal diameter}-\text{Minimal diameter})]*100$$

From these shear stress-FMD curves, the shear stress needed to reach 50% of dilation (EC_50_) and the % of maximal FMD (E_max_) were calculated, in each individual curve.

Experimenters were blinded to groups during data acquisition.

### Immunofluorescence staining (IF)

The day of the sacrifice, n = 3 mice *per* group were anesthetized and then perfused in the heart for 15 min with PBS, followed by 15 min with PFA 4%, for a total of 30 min at 3 ml/min. Brains were removed, put at 4 °C for 24 h in 4% PFA, prior to 24 h in 30% sucrose. They were then frozen in cold 2-methylbutane and stored at −80 °C. Approximately three to four 20 µm slices of hippocampus were cut from brains with a cryostat and put on Superfrost slides. Slides were then fixed for 1 h with PFA 4%, permeabilized 20 min with Triton 0.5%, blocked 1 h with 2% BSA, incubated 48 h at 4 °C with the primary antibody (diluted in 1%BSA) and finally incubated 1 h at room temperature with the secondary antibody (diluted in 1% BSA) and DAPI (1:1000). Each step was inter-stepped with three 5 min washes with PBS. All antibodies used are listed in table S1. Images were acquired with a LSM 710 confocal microscope (Zeiss) using Plan Apochromat 40X/1.3 Oil DIC M27; images are maximum intensity projections created with Z-stack (0.5 µm Z-steps, pinhole 42 µm); lasers in track 1 (405 nm), in track 2 (543 nm), in track 3 (488 nm) and in track 4 (633 nm) were 10%, 10%, 50% and 50%, respectively. Confocal images were analyzed using Image J to delimitate the area of positive cells, divided by the area of DAPI-labeled nuclei (% of total area). In each mouse, 3 brain slices within the hippocampus region were used for IF, and in each slice, 2 different images were taken; data presented are numbers of images after background subtraction. Since n = 3 mice *per* group were used, a total n = 18 images were quantified in each group. Experimenters were blinded to groups during data acquisition.

### Gene expression

RT-qPCR analyses were performed as previously described, in cerebrovascular and in parenchymal fractions [1]. Total RNA was reverse transcribed into first-strand complementary DNA with M-MLV reverse transcriptase (Thermo Fisher Scientific #28,025–021), using random hexamer primers. The qPCR reactions were carried out on diluted RT products by using the DNA-binding dye SYBR Green PCR Master Mix (Applied Biosystem #4,309,155) to detect PCR products with BioRad CFX Real-Time PCR System. The primers of target genes are listed in table S2. All samples were run in duplicate and the fold changes in gene expression were calculated by a ΔΔCT method using the geometric mean of CycloA, HPRT, PPIA and BM2 as housekeeping genes. In order to compare data from different qPCR plates, a common calibrator made of pooled placebo-males (n = 12 mice, for both cerebrovascular and parenchymal fractions) or pooled placebo-females (n = 12 mice) was included in each plate. In each group, 8 mice were randomly selected for quantification.

### Statistical analysis

The data are expressed as means ± standard error of the mean (SEM) of n mice. Group sizes were determined according to our previous studies [1, 39, 41, 58]. For comparisons of means of two groups, unpaired or paired t-tests were adequately performed. To analyze the effect of the single independent factor “age”, within each sex, one-way ANOVA followed by Dunnett’s multiple comparisons tests was performed. For multiple comparisons of means involving a combination of two independent factors such as “sex” and “treatment”, two-way ANOVA followed by post hoc Tukey’s or Sidak’s multiple comparisons tests were performed. P < 0.05 was considered significant. Statistical analyses were performed with the software Prism (Prism 10.0, GraphPad, San Diego, CA, USA).

## Results

### Learning impairment and increased p21 expression with atherosclerosis progression in ATX mice

ATX mice are severely dyslipidemic [40, 42], are more frail and have a shorter life expectancy than their wild-type littermates [1]. Cognitive tests performed at different ages in naïve mice showed that spatial memory (Y Maze) decreased similarly with age in both male and female ATX mice (Fig. 1A). Spatial and recognition memory (NOR) tended to decrease with age in males and decreased in females as soon as 6 months of age (Fig. 1B). Learning memory assessed during the acquisition phase (MWM) evolved differently with age, according to the sex: in males, learning during the 5 successive days clearly slowed at 9- and 12-mo, whereas age had less effect in females (Fig. 1C). In addition, 12-mo female mice learned faster than males (day 2 in males 43 ± 4 *vs.* females 29 ± 6 s, *n* = 15, p < 0.05). Short-term (1 h) memory retention, assessed during probe trials in the MWM test, was unaffected by age or sex (data not shown). Male mice had no delayed (72 h) memory retention, which was only observed in females and not affected by age (Fig. 1D). This suggests that aging female ATX mice display a better delayed memory retention (probe trial), and to a lesser extent, a better learning memory (acquisition phase) than aging male mice. The cerebrovascular expression of the senescence marker p21 (*cdkn1a*), measured by RT-qPCR, tended to increase between the age of 3- to 12-mo by 180% in male (p = 0.092) and increased by 205% in female (*p* < 0.05) ATX mice (Fig. 1E). At both 6- and 9-mo, *p21* expression was higher (*p* < 0.05) in males than in females. Immunofluorescence in the hippocampal region shows that the expression of p21 increased between the age of 6- to 12-mo by 44% in male (*p* = 0.054) and by 88% in female (*p* < 0.05) ATX mice (Fig. 1F).

Based on these data showing learning impairment and increased p21 expression with atherosclerosis progression, male and female ATX mice were treated with the senolytic ABT-263 (Navitoclax) using 2 time frames: a preventive treatment in young mice from 3- to 6-mo, or a curative treatment in middle-aged mice from 9- to 12-mo was applied, to respectively prevent or delay VCI in male and female ATX mice.

### Treatment with ABT-263 was well tolerated

As expected, body weight was smaller in female than in male mice, but it was not affected by ABT-263 in both preventive and curative treatments (Figure S1). Globally, the preventive treatment with ABT-263 (from 3- to 6-mo) had little effect on blood markers of liver or renal function and did not alter the lipid profile or glucose levels, neither in male nor in female mice (**Table S3**). Only in females, ABT-263 lowered alkaline phosphatase. When compared to male-placebo mice, female-placebo mice had lower TG, but higher urea and higher alkaline phosphatase (**Table S3**). Late treatment with ABT-263 (from 9- to 12-mo) lowered cholesterol and TG only in female mice; placebo-female mice had higher glucose, cholesterol, TG and phosphatase alkaline (**Table S3**). These data suggest that, overall, the chronic intermittent treatments with ABT-263 were well tolerated by the mice.

### ABT-263 improves cognition in male ATX mice only

Spatial working memory and exploratory behaviour assessed with the Y maze test were not affected by ABT-263 in young male and female mice (**Figure S2A**). Exploratory behavior decreases strongly with age, and at 12-mo, 100% of the mice failed the Y maze test in all groups (**Figure S2A**); indeed, none of the 12-mo mice, in either group, entered at least 8 times in the arms of the maze (**Figure S2A**).

Recognition memory measured with the NOR test was first validated in young, untreated, 6-mo male ATX mice (n = 23, **Figure S2B**): according to the inclusion criteria of the test, the time spent on both objects was higher than 20 s; during the training trial, the time spent on both objects was similar (p = 0.708) while during the test trial, the time spent on the novel object was significantly higher (p = 0.008), demonstrating that mice discriminate the novel from the familiar object and that the test was functional (**Figure S2B**). In these young, untreated male ATX mice, the recognition index was higher than 50% − the limit for a discrimination by chance − and considered satisfactory (64.9 ± 4.6%; n = 23). The preventive treatment with ABT-263 did not affect recognition memory in young mice of both sexes (**Figure S2C**). Middle-aged mice lacked curiosity for the novel object and ABT-263 did not improve recognition memory, as illustrated by a discrimination index close to or lower than 50% in all 12-mo groups (**Figure S2C**). The success rate for NOR in middle-aged mice was poor, in both males (55%) and females (30%). There was, however, no motor dysfunctions since neither total distance traveled, nor velocity of movement were affected by treatment or sex (**Figure S2D, E**).

We also used the MWM test, a more complex behavioral test performed over 11 days, targeting learning and spatial memory. Because mice must escape from the water, it works even in middle-aged mice lacking exploratory curiosity. Over the 5 days of the learning curve (D1-D5), mice learned to locate the immersed hidden platform and the latency time to reach the target decreased progressively. In young male mice (6-mo), similar learning curves were observed for placebo or ABT-263-treated mice (Fig. 2A). In 6-mo placebo male mice the learning process, performed in the same mice at 3-mo (Fig. 1C), did not reach statistical significance (6-mo placebo D5: 22 ± 5 s *vs.* D1: 33 ± 5 s, p > 0.05), but ABT-263 improved learning in male mice as they successfully located the platform on D4 (6-mo ABT-263 D4: 15 ± 4 s *vs.* D1: 25 ± 3 s, p = 0.0162) (Fig. 2A). In females, placebo- and ABT-263-treated mice exhibited similar learning pathways, they both located the platform on D5 (Fig. 2B). This suggests that ABT-263 was slightly more beneficial in young males, but ineffective in females.

In 12-mo male mice, when compared to placebo, the curative treatment with ABT-263 accelerated the learning process on D4 (p = 0.08) and significantly on D5 (p < 0.05), the latency time to reach the target was twofold shorter in 12-mo ABT-263 (15 ± 4 s) than in 12-mo placebo mice (33 ± 6 s, p < 0.05) (Fig. 2C). In female mice, in contrast, the learning curves were similar between 12-mo placebo (D5: 20 ± 4 s) and 12-mo ABT-263 (D5: 24 ± 4 s) treated groups (Fig. 2D). However, whereas 12-mo placebo female mice started to learn to locate the platform as soon as D2 (12-mo placebo D2: 25 ± 5 s *vs.* D1: 38 ± 4 s, *p* = 0.0109), in 12-mo female mice treated with ABT-263, this learning process was delayed by 3 days (12-mo ABT-263 D5: 24 ± 4 s *vs.* D1: 39 ± 3 s, p = 0.0297) (Fig. 2D). These data suggest that the curative treatment with ABT-263 significantly improved the learning process in middle-aged ATX male mice but was deleterious in females. Mean swimming speed over the 5 days of the learning process was not affected by sex or treatment (**Figure S3**), ruling out any locomotor bias.

One hour after the last test of the learning curve (on D5), the platform was removed and mice were subjected to short-term memory retention probe test: the time spent in the target quadrant (TQ; quadrant where the platform was located) and in the opposite quadrant (OQ) was measured (Fig. 2E, F, G). The same probe test was then repeated 72 h later, to assess delayed memory retention (Fig. 2H, I, J). From these data, a discrimination index for short (1 h) and delayed (72 h) probe tests was calculated (the higher the index, the better memory retention; Fig. 2G, J). In young male mice, ABT-263 significantly improved both short and delayed memory retention (Fig. 2E-G, H-J); in young females, ABT-263 only tended to improve delayed memory retention (*p* = 0.098, Fig. 2J). Of note, short-term discrimination index in 6-mo placebo females tended (p = 0.065) to be higher than in 6-mo placebo males (males: 28 ± 17% *vs.* females: 59 ± 12%) (Fig. 2G), suggesting that there is little room for improvement in females with ABT-263 in prevention; in other words, at 6-mo, placebo female mice tend to have better short-term memory retention than placebo males.

In middle-aged mice, curative treatment with ABT-263 improved (*p* < 0.05) short-term memory retention in males but had no effect in females (Fig. 2F-G). As observed in younger mice, short-term discrimination index in 12-mo placebo females was higher than in 12-mo placebo males (males: 23 ± 18% *vs.* females: 63 ± 8%, *p* = 0.075) (Fig. 2G), suggesting again that there is little room for improvement in females with ABT-263. ABT-263 tended to improve delayed discrimination index (*p* = 0.183) in males, but not in females (Fig. 2J); nevertheless, the time spent in the TQ was significantly different than that in the OQ (*p* < 0.05), only in ABT-263-treated male mice, not in placebo-mice, suggesting significant delayed memory retention with the treatment (Fig. 2I). In contrast, in 12-mo females, whereas in placebo-treated mice TQ time was significantly different than OQ time (*p* < 0.05), in ABT-treated mice, the time spent in TQ was similar than that in OQ (*p* > 0.05) (Fig. 2I), suggesting that ABT-263 was deleterious in females. A significant interaction between the variables “sex” and “treatment” in the 2-way ANOVA of the discrimination index illustrates an opposite effect of ABT-263 in male and female mice: for short-term discrimination index F(1, 54) = 6.700, *p* = 0.0124 and for delayed discrimination F(1, 54) = 4.259, *p* = 0.0439. Mice used to assess the effects of ABT-293 on cognition were tested before and after the treatment, *i.e*. mice were pre-trained, which could affect cognitive responses at the end of the treatment. However, according to our data (not shown), pre-training had very limited effects, if any, on cognitive response to ABT-263 in ATX mice. Altogether, these data suggest that both preventive and curative treatments with ABT-263 improved various aspects of learning and memory retention only in male ATX mice.

### Impact of ABT-263 on atherosclerotic lesions and cerebrovascular flow-mediated endothelium-dependent dilation

**Fig. 3:**
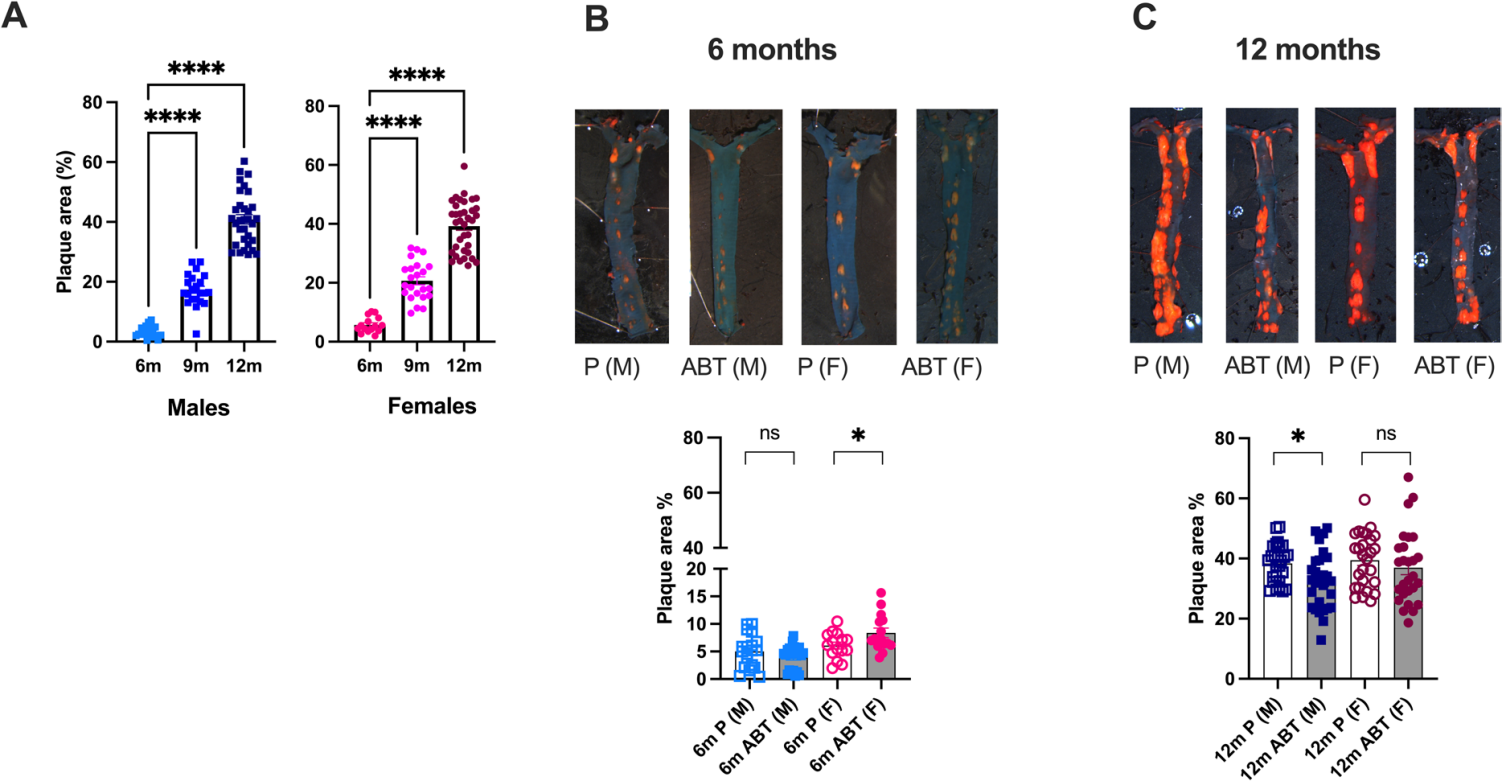
Impact of ABT-263 treatment on atherosclerotic lesion (**A**) Aortic lesion (% area) was quantified by oil-red-O in 6 m (n = 19 males and n = 15 females), 9 m (*n* = 22 males and n = 23 females) and 12 m (*n* = 32 males and *n* = 36 females) ATX mice. *: *p* < 0.05 *vs.* 6 m, one way-ANOVA and Dunnett’s multiple comparisons test. (**B**, **C**) Top panels, typical Oil-Red-O-colored thoracic aortas with atheroma plaques; bottom panels, atherosclerotic plaque expressed as percentage of total aortic area of both males (M) and females (F) mice treated with placebo (P) or ABT-263 (ABT) at 6-month-old (6 m, *n* = 19–21 males, *n* = 17–18 females, **B**) or 12-month-old (12 m, *n* = 26–28 males, *n* = 27 females** C**). *: *p* < 0.05 *vs.* Placebo, Two-way ANOVA (Treatment x Sex) and Sidak’s multiple comparisons test

ATX mice develop spontaneously an aortic atherosclerotic plaque under a regular diet [40, 42]. Cerebral arteries are plaque-free (data not shown). Aortic lesions increased with age, from 6- to 12-mo, similarly in male and female ATX mice (Fig. 3A).

The preventive treatment with ABT-263 had no beneficial effect on aortic lesions in 6-mo males, but in females, ABT-263 paradoxically increased lesion area when compared to placebo (+ 33%, from 7 ± 1 to 9 ± 1%, *p* < 0.05) (Fig. 3B). In contrast, curative treatment with ABT-263 in middle-aged mice had no effect in female mice but slightly reduced the size of the lesion by 15% (from 39 ± 1 to 33 ± 2%, *p* < 0.05) in male mice (Fig. 3C).

**Fig. 4:**
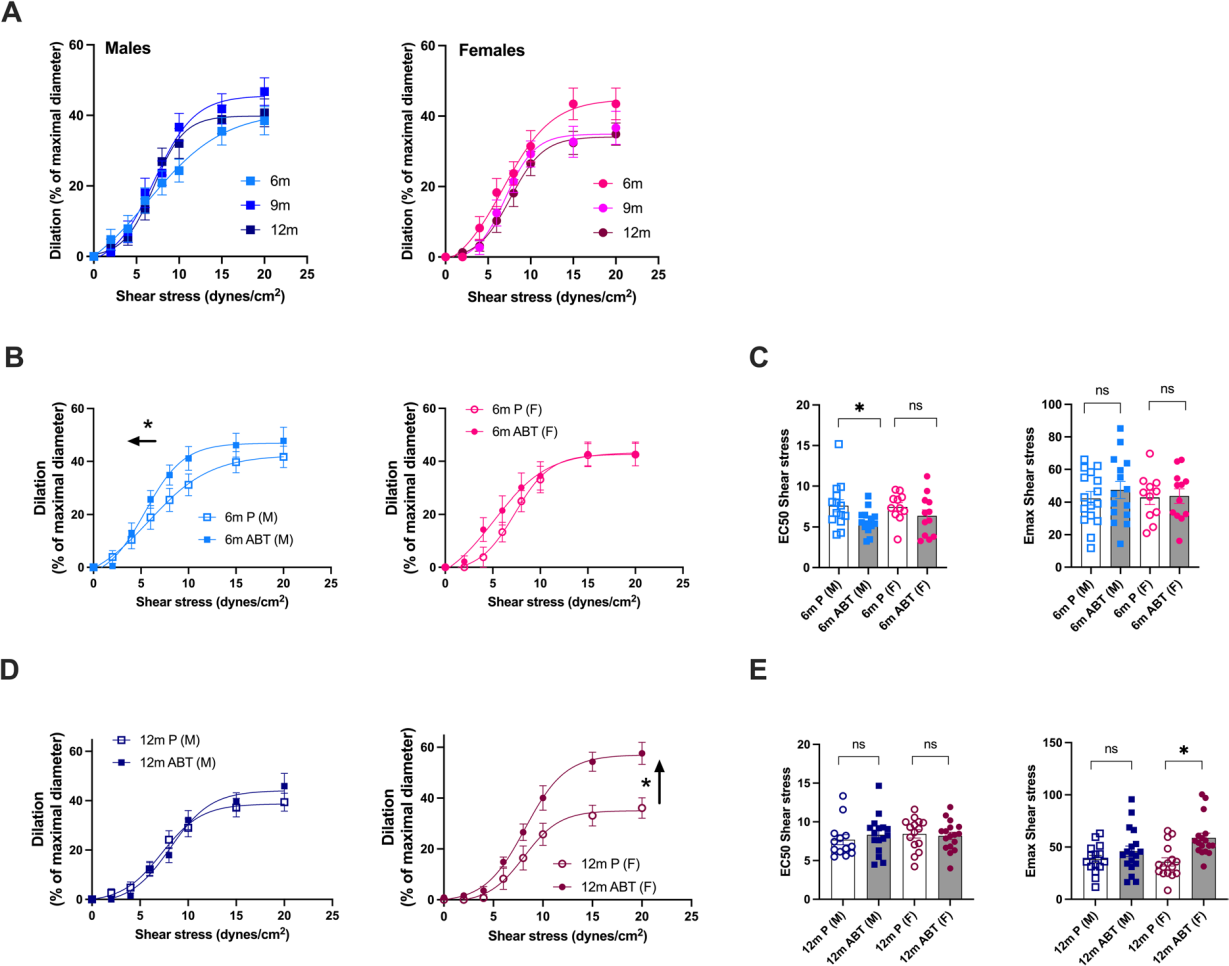
Impact of ABT-263 treatment on cerebral endothelial dilatory function (**A**) Flow-mediated endothelium dependent dilations were measured *ex-vivo* in pressurized pial cerebral arteries from 6 m (*n* = 13 males and *n* = 12 females), 9 m (*n* = 16 males and *n* = 17 females) and 12 m (*n* = 18 males and *n* = 22 females) ATX mice. (**B, C, D, E**) Flow-induced dilation of cerebral arteries of both male (M) and female (F) mice treated with placebo (P) or ABT-263 (ABT) at 6-month-old (6 m, *n* = 16 males, *n* = 11–12 females, **B**) or 12-month-old (12 m, *n* = 15–18 males, *n* = 16–17 females, **D**). EC_50_ (sensitivity to shear stress: the higher the sensitivity, the lower EC_50_) of cerebral arteries of male or female mice treated with placebo or ABT-263; E_max_ (efficacy to shear stress, the higher the efficacy the higher E_max_) of cerebral arteries of male or female mice treated with placebo or ABT-263 at 6-month-old (6 m) (**C**) or at 12-month-old (12 m) (**E**). *: *p* < 0.05 *vs.* Placebo, Two-way ANOVA (Sex x Treatment) and Sidak’s multiple comparisons test. Data are mean ± SEM of n mice

Cerebral endothelium-dependent dilation to shear stress was globally unaffected by age and was similar in males and females (Fig. 4A). In males, the preventive treatment improved endothelium-dependent dilatory responses to shear stress, with no significant beneficial effect in females (Fig. 4B). Indeed, in 6-mo male mice, ABT-263 increased sensitivity to shear stress (*i.e*., decreased EC_50_; from 7.6 ± 0.8 in placebo to 5.7 ± 0.4 dyn/cm^2^ in ABT-263, *p* < 0.05) but not efficacy (no modification of E_max_) (Fig. 4C). In contrast, the curative treatment had no effect in 12-mo males but highly improved efficacy of shear stress on endothelium-dependent dilatory responses in 12-mo females (*i.e.*, increased E_max_ from 36 ± 4 to 59 ± 5%, in placebo and ABT-263, respectively; *p* < 0.05) (Fig. 4D).

Altogether, these data suggest that ABT-263 effects on atherosclerotic plaque size were disconnected from the partial improvement of cerebrovascular endothelial dilatory function, in both male and female mice.

### Opposite sex-dependent effect of ABT-263 on cerebrovascular SASP markers

**Fig. 5:**
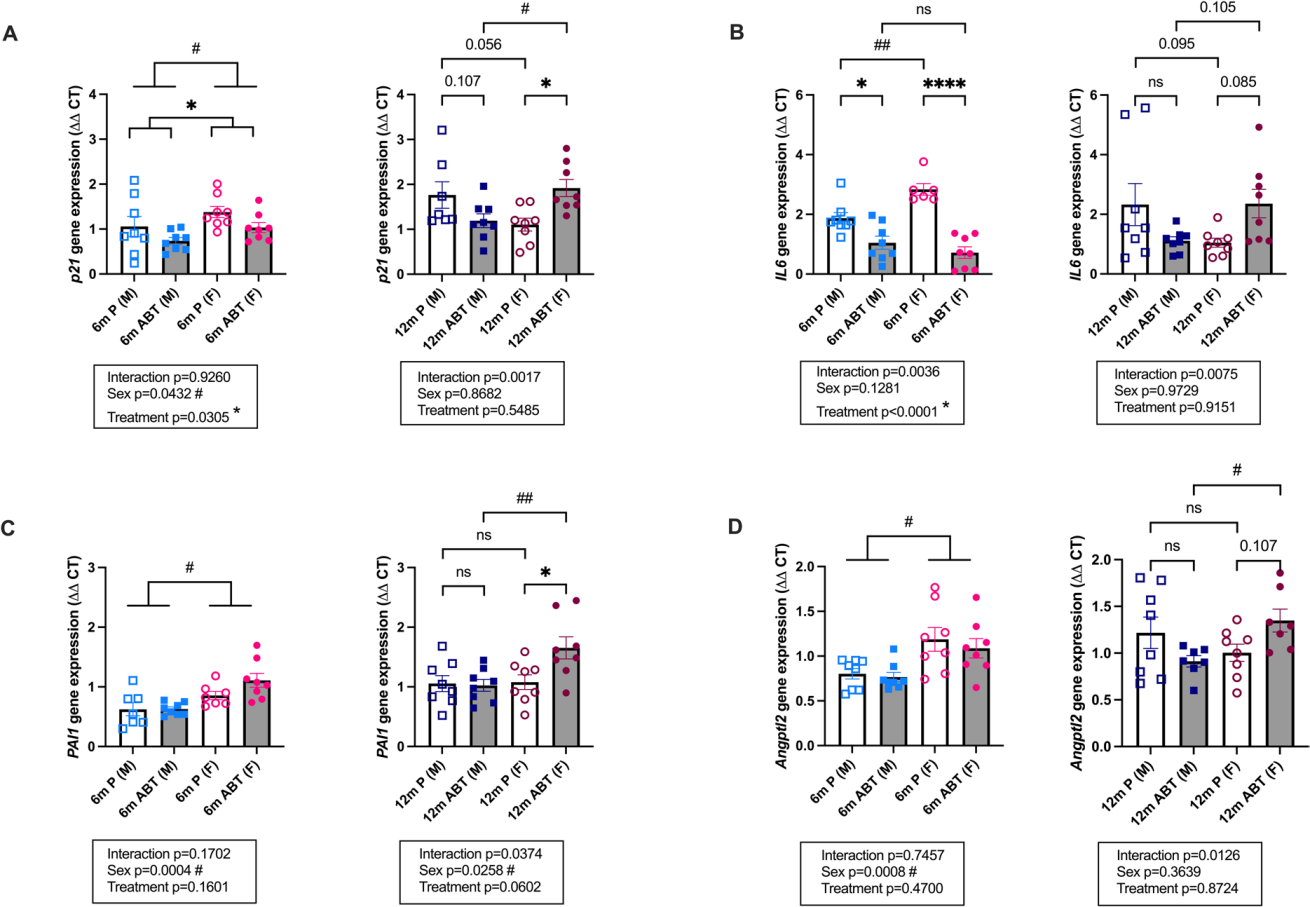
Effect of ABT-263 treatment on gene expression of cerebrovascular p21 and SASP markers. (**A**) *p21* (**B**) *IL6*, (**C**) *PAI1*, and (**D**) *Angptl2* expression in male (M) or female (F) mice treated for 3 months with placebo (P) or ABT-263 (ABT) at 6-month-old (6 m) or 12-month-old (12 m). Data are mean ± SEM of n (n = 7–8) mice. *: *p* < 0.05 *vs.* Placebo, #: *p* < 0.05 vs Males, ANOVA-2way (Sex x Treatment) followed by Sidak’s multiple comparisons. Main sex-effect (#) and main treatment-effect (*) are also indicated when the interaction between the 2 independent factors was not significant. *: *p* < 0.05; ****: *p* < 0.0001; #: *p* < 0.05; ##: *p* < 0.01

It is not clear whether ABT-263 crosses or not the BBB [59], and thus whether the senolytic lowers or not senescence in the brain. We quantified the effects of ABT-263 on the cerebrovascular expression of p21 and different SASP factors (Fig. 5 and Table 1): in young 6-mo mice, preventive treatment with ABT-263 significantly reduced IL6 expression both in male and female mice (Fig. 5), but otherwise had little effect on the SASP markers. However, the cerebrovascular expression of *p21* (a marker of cell cycle arrest [60, 61]), *IL6, PAI1*, *TNFα* and *Angptl2* (4 members of the SASP [60–62]) were higher in females than in males, in placebo and/or ABT-263 groups (Fig. 5 and Table 1), highlighting a sexual dimorphism. Indeed, a main sex-effect was observed for these markers in young mice: *p21* (F(1, 28) = 4.485, *p* = 0.0432), *PAI1* (F(1, 26) = 16.19, *p* = 0.0004), *Angptl2* (F(1, 28) = 14.10, *p* = 0.0008), *TNFα* (F(1, 26) = 14.75, *p* = 0.0007) (Fig. 5 and Table 1).

**Table 1:** Impact of preventive (from 3 to 6-mo) and curative (from 9 to 12-mo) ABT-263 treatment on gene expression in the cerebrovascular fraction (data are 2^-ΔΔCT)

**Preventive** **3–6 m**	**Males** **Placebo**	**Males** **ABT-263**	**Females** **Placebo**	**Females** **ABT-263**
*TNFα*	0.276 ± 0.019 (7)	0.374 ± 0.111 (8)	0.655 ± 0.074 (7)	1.613 ± 0.370 (8) * #
*ICAM1*	0.936 ± 0.085 (6)	0.700 ± 0.0081 (8)	1.033 ± 0.097 (8)	0.751 ± 0.074 (8) *
*Occludin*	0.888 ± 0.111 (8)	0.865 ± 0.081 (8)	0.815 ± 0.082 (7)	0.759 ± 0.105 (8)
*Claudin-5*	1.088 ± 0.090 (8)	1.878 ± 0.374 (8)	1.015 ± 0.176 (8)	0.894 ± 0.264 (8) #
**Curative** **9–12 m**	**Males** **Placebo**	**Males** **ABT-263**	**Females** **Placebo**	**Females** **ABT-263**
*TNFα*	0.899 ± 0.083 (8)	1.396 ± 0.198 (8) *	0.803 ± 0.103 (7)	1.225 ± 0.126 (8)
*ICAM1*	0.809 ± 0.140 (7)	0.767 ± 0.110 (8)	0.882 ± 0.075 (8)	1.115 ± 0.112 (8)
*Occludin*	0.866 ± 0.080 (7)	1.109 ± 0.076 (7)	0.794 ± 0.073 (8)	1.008 ± 0.097 (8)
*Claudin-5*	0.751 ± 0.099 (7)	0.841 ± 0.102 (8)	0.801 ± 0.135 (8)	0.980 ± 0.187 (8)

Data are mean ± SEM of n mice. ANOVA-2way (Sex x Treatment) and Sidak’s multiple comparisons test (*p* < 0.05 *: placebo *vs.* ABT-263, treatment effect; *p* < 0.05 #: Male *vs.* female, sex effect)

At 12-mo, ATX mice exhibit an increased BBB permeability previously demonstrated by Evans blue extravasation and by the presence of microbleeds [1], which could permit diffusion of ABT-263 into the parenchyma [50]. In the cerebrovascular fraction, gene expression of markers of senescence-associated damage tended to decrease in 12-mo males (*p21,* −32% *p* = 0.107; *IL6,* −52% *p* = 0.116, *Angptl2,* −25% *p* = 0.166;) (Fig. 5), without significant impact on the parenchymal fraction (Table 2). In contrast, in 12-mo females, ABT-263 paradoxically increased *p21* (*p21,* + *73%* *p* < 0.05) and SASP factors of inflammation in the cerebrovascular fraction (*PAI1,* + *53%* *p* < 0.05*, IL6,* + 125% *p* = 0.085, and *Angptl2* + 34% p = 0*.*107) (Fig. 5); ABT-263 also increased two recognized markers of senescence in the parenchymal fraction in females (*p21* + 302%*,* *p* < 0.05 and *LMNA*, + 40% *p* = 0.058) (Table 2). Altogether, these data suggest an opposite sex-dependent effect of ABT-263 on cerebrovascular p21 and SASP factors, illustrated by significant interactions between the two independent variables “sex” and “treatment”: vascular *p21* (F(1, 27) = 12.22, p = 0.0017), *Angptl2* (F(1, 26) = 7.176, p = 0.0126), *PAI1* (F(1, 28) = 4.776, *p* = 0.0374), *IL6* (F(1, 28) = 8.297, *p* = 0.0075) (Fig. 5), and parenchymal *p21* (F(1, 28) = 6.55, *p* = 0.0162) and parenchymal *LMNA* (F(1, 27) = 7.170, *p* = 0.0125) (Table 2). The rise in p21 and SASP markers in 12-mo ABT-263-treated female ATX mice could, at least partially, be associated with the absence of improved cognition in female mice. Of note, in contrast to *p21*, the senescence marker *p16* was not detectable in the brain of ATX mice, in any of the groups, neither at the gene (cerebrovascular or parenchymal fractions), nor at the protein level (hippocampal region) (data not shown).

**Table 2:** Impact of preventive (from 3 to 6-mo) and curative (from 9 to 12-mo) ABT-263 treatment on gene expression in the parenchymal fraction (data are 2^-ΔΔCT)

**Preventive** **3–6 m**	**Males** **Placebo**	**Males** **ABT-263**	**Females** **Placebo**	**Females** **ABT-263**
*p21*	1.120 ± 0.155 (6)	1.127 ± 0.110 (8)	1.193 ± 0.142 (8)	1.288 ± 0.243 (8)
*Angptl2*	0.973 ± 0.076 (7)	0.603 ± 0.135 (8)	1.223 ± 0.150 (7)	1.129 ± 0.162 (8) #
*LMNA*	0.997 ± 0.082 (6)	0.996 ± 0.053 (8)	1.041 ± 0.090 (8)	1.092 ± 0.066 (8)
*TNFα*	1.061 ± 0.156 (6)	1.536 ± 0.469 (8)	1.412 ± 0.334 (8)	2.478 ± 0.515 (8)
*IL-6*	2.725 ± 1.231 (6)	1.313 ± 0.496 (5)	1.811 ± 0.574 (6)	1.486 ± 0.725 (8)
*GFAP*	0.881 ± 0.063 (6)	1.032 ± 0.051 (8)	1.073 ± 0.092 (7)	0.947 ± 0.119 (8)
*Iba-1*	0.935 ± 0.049 (6)	0.718 ± 0.070 (8)	0.952 ± 0.050 (8)	1.249 ± 0.086 (8) * #
*Synaptophisin*	0.899 ± 0.086 (6)	1.005 ± 0.057 (8)	0.767 ± 0.140 (8)	0.908 ± 0.126 (8)
*NeuN*	1.348 ± 0.138 (6)	0.939 ± 0.057 (8)	1.335 ± 0.146 (8)	1.120 ± 0.143 (8)
*BDNF*	0.856 ± 0.109 (6)	0.867 ± 0.110 (8)	1.232 ± 0.125 (8)	1.254 ± 0.186 (8)
**Curative** **9–12 m**	**Males** **Placebo**	**Males** **ABT-263**	**Females** **Placebo**	**Females** **ABT-263**
*p21*	1.034 ± 0.137 (8)	1.429 ± 0.214 (8)	1.111 ± 0.105 (8)	4.464 ± 1.122 (8) * #
*Angptl2*	0.984 ± 0.111 (8)	0.939 ± 0.099 (8)	0.831 ± 0.023 (8)	0.998 ± 0.070 (8)
*LMNA*	1.065 ± 0.162 (8)	0.852 ± 0.066 (8)	0.859 ± 0.038 (7)	1.203 ± 0.093 (8)
*TNFα*	0.923 ± 0.147 (8)	1.155 ± 0.149 (7)	1.297 ± 0.110 (8)	1.329 ± 0.142 (7)
*IL-6*	0.978 ± 0.192 (8)	0.954 ± 0.213 (8)	1.715 ± 0.315 (8)	0.773 ± 0.188 (8) *
*GFAP*	1.165 ± 0.134 (8)	1.147 ± 0.075 (8)	1.046 ± 0.075 (8)	1.146 ± 0.098 (8)
*Iba-1*	0.963 ± 0.076 (8)	0.883 ± 0.078 (7)	1.158 ± 0.028 (8)	1.250 ± 0.055 (8) #
*Synaptophisin*	1.140 ± 0.089 (8)	0.936 ± 0.087 (8)	1.482 ± 0.205 (7)	1.355 ± 0.124 (8) #
*NeuN*	1.088 ± 0.071 (8)	1.133 ± 0.118 (8)	1.085 ± 0.142 (8)	1.248 ± 0.095 (8)
*BDNF*	0.868 ± 0.099 (8)	0.754 ± 0.070 (7)	1.138 ± 0.089 (8) #	0.952 ± 0.068 (8)

Data are mean ± SEM of n mice. ANOVA-2way (Sex x Treatment) and Sidak’s multiple comparisons test (*p* < 0.05 *: placebo *vs.* ABT-263, treatment effect; *p* < 0.05 #: Male *vs.* female, sex effect)

### Opposite sex-dependent effect of ABT-263 on endothelial cells density

**Fig. 6:**
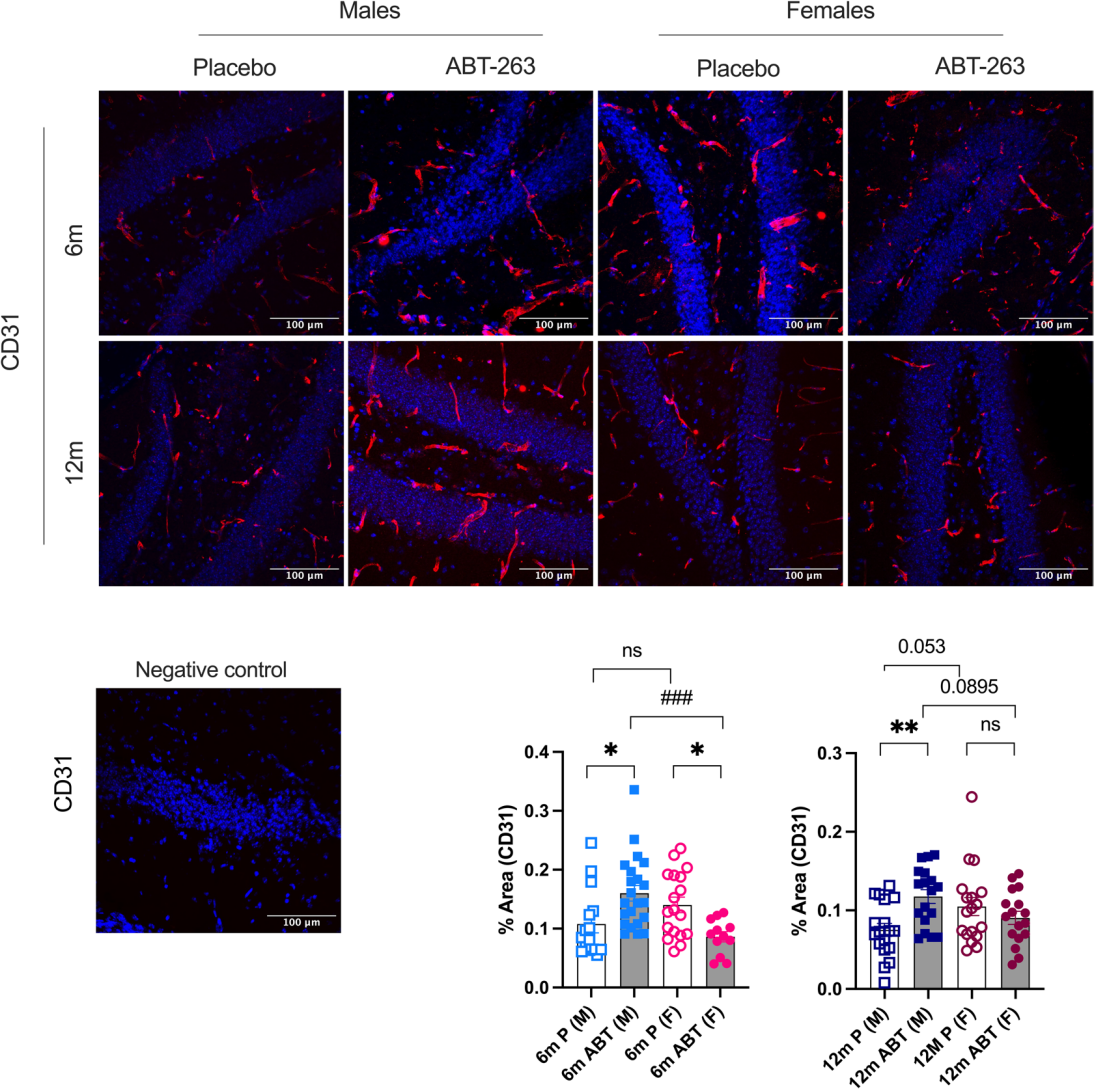
Effect of ABT-263 treatment on hippocampal protein marker of endothelial cells. Immunostaining in the hippocampus of CD31, a marker of endothelial cells in males (M) and females (F) ATX mice treated with placebo (P) or ABT-263 for 3 months (ABT), at 6-month-old (6 m) or 12 month-old (12 m). Typical images and a negative control for each staining are shown; scale bar = 100 µm, for all images. Data are mean ± SEM of n (n = 15–18) images (% area of positive staining / % area of DAPI-labelled nuclei). *: *p* < 0.05 vs Placebo, #: *p* < 0.05 vs Males, ANOVA-2way (Sex x Treatment) followed by Sidak’s multiple comparisons. *: *p* < 0.05; **: *p* < 0.01; ###: *p* < 0.001

Both preventive and curative treatments with ABT-263 increased CD31-positive endothelial cells density in the hippocampus area of male mice; in females, ABT-263 had the opposite effect in young mice and no effect in middle-aged mice (Fig. 6). Cerebrovascular *ICAM1* mRNA expression was also significantly decreased (*p* < 0.05) by ABT-263 in 6-mo females (Table 1). In addition, the expression of *claudin-5* coding for a tight junction at the BBB, was increased by ABT-263 in young 6-mo male mice (*p* = 0.065), but significantly decreased (*p* < 0.05) in females: main sex-effect for *claudin-5* (F(1, 28) = 4.496, *p* = 0.0430). In middle-aged mice of both sexes, ABT-263 had no impact on *claudin-5* or *occludin* (Table 1). Altogether, these data suggest a beneficial effect of ABT-263 on endothelial cells markers in male mice, but not in females.

### Opposite sex-dependent effect of ABT-263 on astrocytes and glial cells activation

**Fig. 7:**
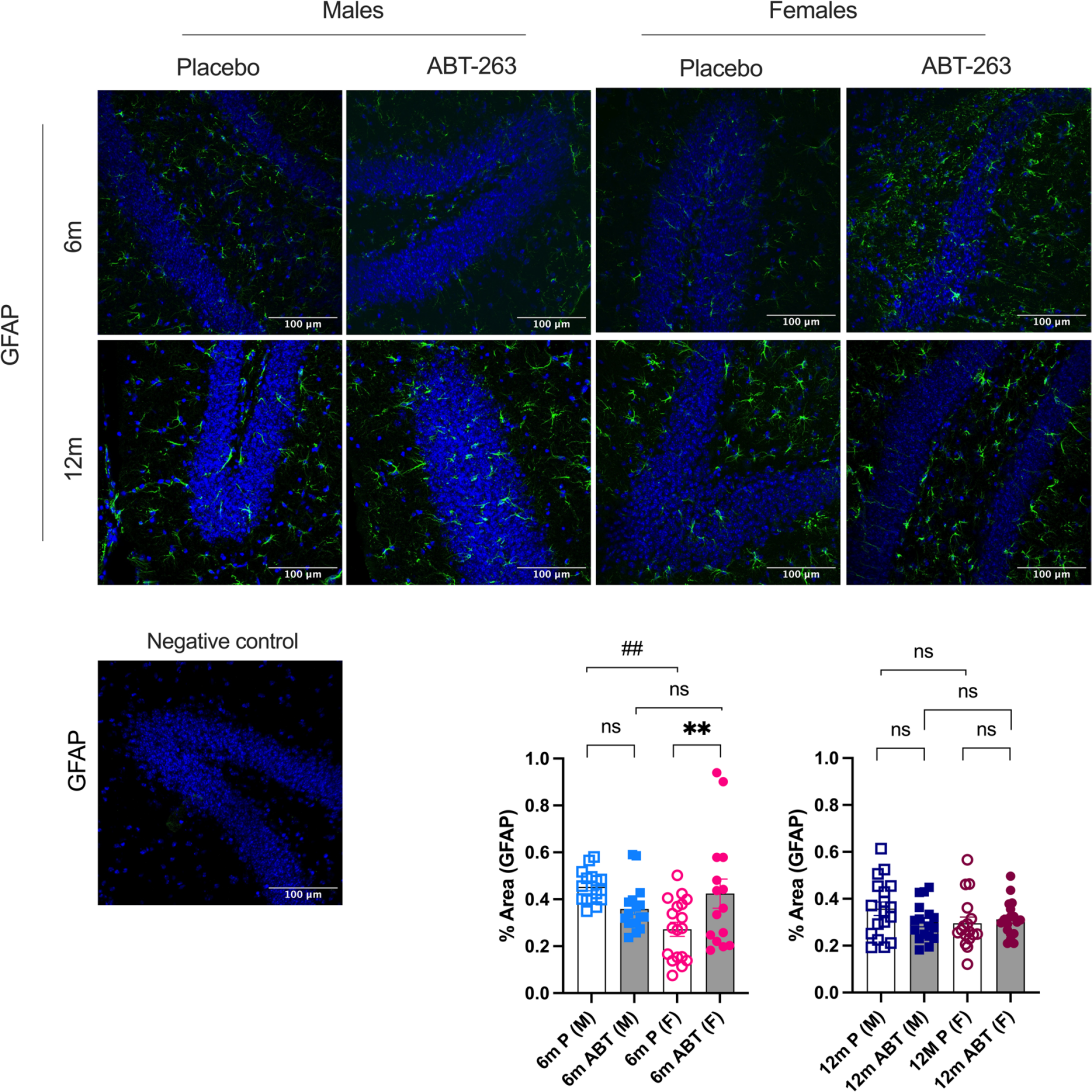
Effect of ABT-263 treatment on hippocampal protein marker of astrogliosis. Immunostaining in the hippocampus of GFAP, a marker of astrocytes in males (M) and females (F) ATX mice treated with placebo (P) or ABT-263 for 3 months (ABT), at 6-month-old (6 m) or 12 month-old (12 m). Typical images and a negative control for each staining are shown; scale bar = 100 µm, for all images. Data are mean ± SEM of n (*n* = 15–18) images (% area of positive staining / % area of DAPI-labelled nuclei). *: *p* < 0.05 vs Placebo, #: *p* < 0.05 vs Males, ANOVA-2way (Sex x Treatment) followed by Sidak’s multiple comparisons. **: *p* < 0.01; ##: *p* < 0.01

In agreement with an opposite sex-effect on cognition in ABT-263-treated mice, astrocyte activation (GFAP) tended to be reduced in males at 6-mo (*p* = 0.133) and 12-mo (*p* = 0.171) but astrogliosis was significantly increased in young females (Fig. 7). Parenchymal *GFAP* gene expression did not change significantly, highlighting a discrepancy between protein and mRNA expression of GFAP in 6-mo females (Table 2).

**Fig. 8:**
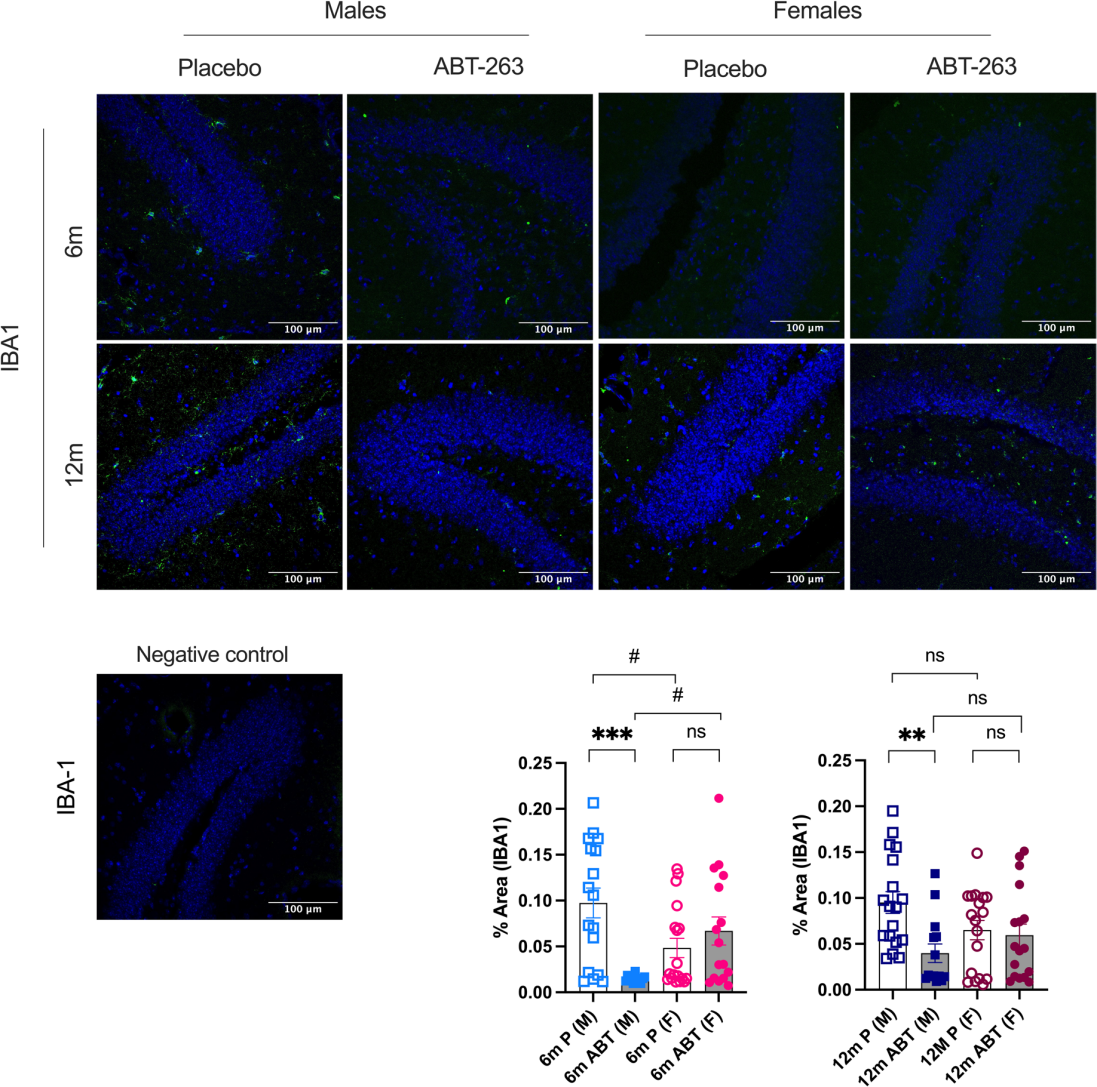
Effect of ABT-263 treatment on hippocampal protein marker of gliosis. Immunostaining in the hippocampus of IBA1, a marker of glial cells in males (M) and females (F) ATX mice treated with placebo (P) or ABT-263 for 3 months (ABT), at 6-month-old (6 m) or 12-month-old (12 m). Typical images and a negative control for each staining are shown; scale bar = 100 µm, for all images. Data are mean ± SEM of n (*n* = 15–18) images (% area of positive staining / % area of DAPI-labelled nuclei). *: *p* < 0.05 vs Placebo, #: *p* < 0.05 vs Males, ANOVA-2way (Sex x Treatment) followed by Sidak’s multiple comparisons. **: *p* < 0.01; ***: *p* < 0.001; #: *p* < 0.05

In addition, IBA1-positive glial cells decreased significantly only in male ABT-263-treated mice, at both ages (Fig. 8), together with lower brain parenchymal *Iba1* gene expression in males than in females mice: in 6-mo mice, main sex-effect for *Iba1* (F(1, 26) = 16.04, *p* = 0.0005) and in 12-mo mice, main sex-effect for *Iba1* (F(1, 27) = 20.82, *p* < 0.0001) (Table 2). This suggests decreased microgliosis in ABT-263 treated males. Although IBA1 protein expression was not affected by ABT-263 in young and middle-aged female mice (Fig. 8), *Iba1* gene expression was significantly increased by the treatment at both ages in females (Table 2); with these discrepancies between protein and mRNA expression of IBA1, our data suggest either no benefit or increased microgliosis in ABT-263 treated females.

Synaptophysin is a pre-synaptic protein that reflects synaptic density. Better cognition in male mice treated with ABT-263 is not matched by an increased synaptic density, as RT-qPCR data in brain parenchymal fraction (in 12-mo mice, not observed in young mice) show that *synaptophysin* expression was higher in 12-mo ABT-263 females than males, main sex-effect in 12-mo for *synaptophysin* (F(1, 27) = 8.638, *p* = 0.0067) (Table 2). Similarly, brain parenchymal *BDNF* expression was higher in 12-mo female mice than in males, main sex-effect for *BDNF* (F(1, 27) = 7.829, *p* = 0.0094) (Table 2). However, expression of the neuronal marker *NeuN* was not different between groups (Table 2). Altogether, higher *Synaptophysin* and *BDNF* expression may reflect a potentially better “memory reserve” in females. Concomitant higher levels of parenchymal inflammatory *IL6* (*p* = 0.066), *TNFα* (p = 0.111) and *Iba1* (*p* = 0.061) in 12-mo placebo-female mice than in males (Table 2), may have compromised the effect of ABT-263 on cognition in females.

## Discussion

Our data show, globally, that male atherosclerotic mice responded positively to the senolytic treatment but not female mice. In young male ATX mice, prevention with ABT-263 improved short-term (1 h) and delayed (72 h) memory retention. This was associated with a higher endothelial sensitivity to shear stress and a higher vascular CD31^+^ endothelial cell density in the hippocampal area – the brain region most responsible for memory. Lower activation of both astrocytes (GFAP) and glial cells (IBA1) in male-treated mice may also contribute to the beneficial effects of ABT-263 on cognition. However, ABT-263 had no impact on plaque size, p21 and SASP markers in young ATX mice, suggesting that beneficial effects of the senolytic on cognition may be indirect. In young female mice, the preventive treatment tended to improve delayed memory retention; however, atherosclerotic plaque was magnified, endothelial function was unaffected, hippocampal staining of GFAP increased and expression of vascular CD31^+^ endothelial cells decreased. Hence, unlike in males, the treatment appears deleterious between 3- and 6-mo in female ATX mice.

In middle-aged male mice, the curative treatment clearly improved the learning process over the 5 days of the MWM, as well as both short- and delayed memory retention, suggesting that age-dependent cellular senescence-associated damage negatively impacts cognition in atherosclerotic male mice. The beneficial cognitive effect of ABT-263 in these middle-aged male mice was associated with a higher density of vascular CD31^+^ endothelial cells, and a lower activation of glial cells (IBA1). ABT-263 also decreased the expression of several vascular SASP markers in male mice, but increased SASP expression in females. In the latter, this rise in senescence induced by ABT-263 was associated with a delayed learning process, in disconnect with the observed increase in maximal endothelium-dependent dilation. Altogether, these data demonstrate a sexual dimorphism in response to a senolytic treatment (either preventive or curative) on cognitive functions in atherosclerotic mice.

### Vascular contribution to cognitive impairment

In the literature, the impact of senescence in brain cells on cognition was reported in different murine models, including Tau-dependent [14] and AD [63] neurodegenerative disease models, after traumatic brain injury [18, 23], irradiation or chemotherapy [19, 28, 64] or in the context of aging [25, 31, 35, 36, 38, 49]. All studies reported prevention or restauration of cognitive functions by a senolytic treatment such as ABT-263, fisetin or the combination of dasatinib and quercetin (D + Q). These data strongly suggest causality for cellular senescence in the initiation and progression of cognitive decline via neuroinflammation and possibly neurovascular uncoupling in these various models. To the best of our knowledge, our report is the first to demonstrate a beneficial effect of ABT-263 in atherosclerosis-dependent VCI. However, despite a significant faster learning process in ABT-263-treated middle-aged male ATX mice, endothelial dysfunction assessed in pial arteries was not corrected; inversely, despite a significant improvement of FMD in ABT-263-treated middle-aged female ATX mice, cognition was not improved. In the literature, some studies suggested that cellular senescence contributes to macrovascular endothelial aging, as ABT-263 [65], D + Q [34], and fisetin [33] improved carotid endothelium-dependent relaxations (EDR) in aged mice; in these studies, cognitive functions were not assessed. However, a link between cerebrovascular function and cognition (*i.e*. neurovascular coupling) should only be established at the microvascular level; evaluation of macrovascular pial endothelial function cannot be extrapolated to microvascular vessels. A causal relationship between senescence, microvascular endothelial and neurovascular dysfunction was established in mice exposed to chemotherapy (paclitaxel) treated with ABT-263, that improved in vivo endothelium-dependent neurovascular coupling and cognitive performances [28]. It has also been reported that ABT-263 improved neurovascular coupling and learning without improving aorta EDR in aged mice [31]. In the present study, atherosclerosis-dependent cognitive impairment in 12-mo ATX mice can be caused by both macrovascular (atherosclerosis-induced decreased CBF and carotid stiffening previously reported by us [39, 41]) and by microvascular (atherosclerosis-induced increased BBB permeability and NVU uncoupling [1]) dysfunctions, although not measured in the present study. In our study, the measure of ex vivo FMD in pial cerebral arteries does not reflect the complexity of in vivo cerebral perfusion and does not permit to establish a direct link between cerebrovascular function and cognition. Complexity further increases with the demonstration that cerebrovascular responses to ABT-263 are sex-dependent.

### Sexual dimorphism

ABT-263 improved cognition solely in male mice and was ineffective or deleterious in females. Three recent studies reported similar sex-dependent responses to senolysis. The first showed that chronic fisetin treatment rescued age-associated memory decline (MWM and NOR tests) only in adult 13-mo male C57BL/6 mice [35]. The authors also used D + Q: it had limited effects in males, but this senolytic cocktail was deleterious in 13-mo females, with increased SASP expression and reduced cognitive performances [35]. The second study reported that ABT-263 reduced senescence only in aged male mice with traumatic brain injury, improving memory (MWM) in male mice only [23]. Recently, Rani et al. showed that senolytic treatment with either D + Q or ABT-263 failed to improve age-related cognitive decline in 19-mo female rats [49], in contrast to the beneficial effects observed with similar treatments in males rats [38]. The authors proposed that C57BL/6 male mice may age faster than females, or in other words that females maintain a better cellular health throughout their life, explaining why only males benefited from senolysis [35]. Likewise, Schwab and colleagues proposed that female mice might have a higher tolerance for genotoxic stress than males, explaining why ABT-263 was only beneficial in males [23]. Rani et al. proposed that because in aged female rats, the decrease of sex hormones such as estradiol was not compensated by the senolytic treatment, it is possible that estradiol − protective against senescence − also acts independently of cellular senescence [49]. Interestingly, a recent study reported that ABT-263 accelerated ovarian aging in older female mice [66]. Therefore, the potential role of hormonal factors, such as estrogen, on the sexual dimorphism observed in response to a senolytic treatment clearly deserves further investigations.

We report a better short-term memory retention in young (6-mo) and middle-aged (12-mo) placebo female ATX mice than in age-matched placebo males, which could partially explain why ABT-263 did not (further) increase short-term memory in females. We also report a lower GFAP and IBA1 immunostaining in young female placebo mice than in age-matched placebo males, which could also contribute to the absence of beneficial effects of ABT-263 in females. In association with a higher short-term memory retention in middle-aged placebo-female mice, we also report higher *BDNF* expression in placebo-females than in placebo-males. This could suggest, in agreement with Fang and colleagues [35] and Schwab and colleagues [23], and with our recent data in patients with severe coronary artery disease [67], that female cells are more resilient and resistant to stress. It is also possible that healthier female cells, but not males, use senescence pathways as defense mechanisms, explaining the deleterious effects of ABT-263 in female ATX mice. Whether this healthier status in female cells is related to protective senescence and/or linked to hormonal status remains to be demonstrated. Our data suggest that in males ATX mice, treatment with a senolytic reduced p21 and SASP factors and improved cognition, suggesting that senescent cells contribute to cognitive decline; in females, ABT-263 increased SASP markers and worsened cognitive functions, suggesting another, yet unknown, defense/repair role for senescence in female cells.

### Which cells are senescent in the brain and associated with cognitive decline?

Vascular senescence was assessed by p21 expression and some SASP factors (IL6, TNFα, PAI1, Angptl2); however, our study lacks direct evidence of vascular senescence and does not permit to determine which brain cell types are senescent and contribute to VCI in ATX male mice. We found that both GFAP-positive astrocytes, CD31-positive endothelial cells, and IBA-1-positive microglial cells were sensitive to ABT-263, at least in males. Senescent astrocytes, the most important and abundant cells in the brain that maintain the function of the synapses, have been described in many studies and various mouse models to contribute to cognitive impairment [14–16, 19]. In addition, microglial cells that function as immune cells in the brain [68], are very vulnerable in the aging brain and highly susceptible to senescence [69]. Single-cell RNA sequencing approaches identified senescent microglial cells in various murine models with cognitive deficits [21, 22], but also senescent endothelial cells [28–30], and senescent neurons [23, 26]. Thus, any cell type from the brain circulation (pericytes, endothelial and smooth muscle cells), and the parenchyma (astrocytes, microglia and neurons) can become senescent and contribute to the initiation and the progression of neurovascular uncoupling and thus, ultimately, promote abnormal neuronal activity and cognition.

### What is the optimal timing for senolytic treatment?

Using the MWM test, the beneficial effect of the senolytic was more obvious in middle-aged male mice, when the treatment was started once the cognitive defects were established: treatment from 9 to 12-mo clearly accelerated the learning process, significantly increased short and delayed memory retention, and tended to decrease p21 and SASP expression in males while having opposite effects on cognition and p21/SASP factors in females. In 6-mo younger male mice, that already exhibit higher cerebrovascular p21-senescence than 3-mo mice, preventive ABT-263 treatment increased short- and delayed memory retention. Altogether, this suggests that the senolytic was able to both prevent and delay cognitive decline in ATX, at least in male mice. Nelke and colleagues asked at what time point does the cellular senescent load become deleterious to the brain [52]? It is not clear whether there is a threshold beyond which the accumulation of senescent cells impacts organ functions [53], but our data suggest that senescent cells contribute to the cognitive decline of male ATX mice. Importantly, they also demonstrate that in female ATX mice, senolysis may be deleterious, suggesting that female cells may use senescence as a defence mechanism.

### Clinical relevance

Increased senescence markers in prefrontal cortex of patients with Alzheimer disease, in microglial cells, astrocytes and neurons, have been reported [17, 26]. Recently, clinical data showed that senolytics could potentially alleviate human brain aging: a first phase I clinical trial of D + Q administration in symptomatic patients with Alzheimer disease has been conducted to assess central access, efficacy and safety of the senolytic treatment [70]. This pilot study (SToMP-AD; NCT04685590) showed evidence of brain penetrance of D (not Q), and reports a good tolerance of the treatment; baseline to post-treatment changes in cognitive functions were not observed, due to small sample size and short duration of the treatment [70]. Of note, significantly higher GFAP and IL6 levels were observed in the cerebrospinal fluid from D + Q treated patients, reflecting either clearance of senescent astrocytes or an acute inflammatory response of the treatment [70]. Another clinical trial (ALSENLITE; NCT04785300) targeting senescence with D + Q in patients with mild cognitive impairment has been described [71], but the results are not yet available. Patients with severe COVID-19 may also exhibit neurological disorders, and a significant accumulation of senescent cells was noted in the brain of infected patients; these senescent cells may promote inflammatory neuropathologies and could be sensitive to senotherapies [72]. None of these studies, however, addresses the sex-dependent drug response. Together with previous reports [23, 35, 49], our current data would recommend a strict follow-up of female patients included in senolytic drug trials.

### Limitations of the study

First, there is no universal cell-specific marker of senescence, and to demonstrate the presence of senescent cells, the detection of multiple markers is usually necessary. In the present study, the senescent marker p21 (but not p16, which was undetectable), and the SASP factors IL6, TNFα, PAI1, and Angptl2 non-specifically identified the presence of senescent cerebrovascular and brain cells. Only the detection of multiple senescence markers by flow cytometry in labelled cerebrovascular cells, and/or single-cell RNA sequencing has the potential to identify the senescent cell type, and the various senescent genes and pathways associated. Second, the measure of FMD in isolated pial cerebral arteries does not reflect the in vivo complexity of the cerebrovascular biology that integrates both macrovascular and microvascular beds. Direct in vivo assessments of CBF and neurovascular coupling during a hyperemic stimulus would complement our endothelial function data. Third, the molecular mechanism underlying the sexual dimorphism in the beneficial (males) or deleterious (females) cognitive effect of ABT-263 in ATX mice has not been investigated; evaluation of the hormonal role would be particularly relevant. Fourth, lack of age-matched wild-type controls may hinder the interpretation of cognitive and vascular impairments in the ATX model. However, because young and middle-aged wild-type mice are cognitively alert compared to ATX mice [1], they may not express significant levels of markers of vascular damage/senescence and would not respond to ABT-263 or exhibit off target effects. Finally, mice used to assess the effects of ABT-293 on cognition were tested before and after the treatment, *i.e*. mice were pre-trained, which could affect cognitive responses at the end of the treatment. However, according to our data, pre-training had very limited effects on cognitive response to of ABT-263 in ATX mice.

### Future directions

Our data globally suggest that the senolytic ABT-263 has an opposite, sex-dependent effect in the cognitive function of atherosclerotic mice. Future studies using single-cell RNA sequencing will identify the various molecular pathways that trigger atherosclerosis-dependent senescence, inflammation, damage/repair associated with this dimorphism. The possibility of combining spatial transcriptomics could be of high interest as recently reviewed [73]. This would help understanding the molecular basis of the sexual dimorphism, including metabolic and hormonal pathways. This single cell and spatial transcriptomic would benefit from a sensitive method to assess local CBF (and thus to the microvasculature) such Ultrasound Localization Microscopy (ULM) [74, 75]. Combining spatial CBF changes to spatial transcriptomic at the single cell level would be of tremendous interest in our model of atherosclerosis-mediated VCI. Finally, one important missing information is the kinetic of VCI development: to clearly understand the decline in the cognitive evolutionary status of these ATX mice and the sex-related differences, the weakest molecular link and cell type that first trigger cognitive decline need to be identified, to inform on the adequate time for treatment initiation, with ABT-263 or other senolytics. Testing additional compounds would indeed strengthen conclusions about the role of senescence in VCI.

In conclusion, we show a sex-dependent effect of the senolytic ABT-263 on cognition in atherosclerotic mice, with a beneficial cognitive effect seen only in males. Both preventive and curative treatment with ABT-263 improved learning and retention memory in male ATX mice, suggesting a contribution of senescent cell load in the brain on cognitive dysfunction. Importantly, the deleterious effects of ABT-263 in female ATX mice recommend caution in future trial; based on the literature, senolytics could be expected to be positive in men and deleterious in women.

## Supplementary Information

Below is the link to the electronic supplementary material.

Supplementary file1 (DOCX 9879 KB)
