# Supplementary material for: The senolytic ABT-263 improves cognitive functions in middle-aged male, but not female, atherosclerotic LDLr−/−;hApoB100+/+ mice
Source: GeroScience. 2025 Feb 21;47(3):4577–600. doi: 10.1007/s11357-025-01563-3 (PMC12181456; doi:10.1007/s11357-025-01563-3)
Supplement: Supplementary file 1 — Supplementary file1 (DOCX 9879 KB) [file 11357_2025_1563_MOESM1_ESM.docx]

**Cerebrovascular senescence plays an opposite sex-dependent role in the cognitive function of atherosclerotic mice**

Mélanie Lambert^1,2^, Géraldine Miquel^2^, Louis Villeneuve^2^, Nathalie Thorin-Trescases^2^, Eric Thorin^2,3^

**Supplemental data:**

**Table S1** List of primary and secondary antibodies

**Table S2** List of primers used for real-time quantitative PCR

**Table S3** Impact of preventive (from 3-6m) and curative (from 9-12m) senolytic treatment on blood markers

**Figure S1** Mice body weight follow-up

**Figure S2** Cognition: Y Maze and NOR tests

**Figure S3** Total mean speed of swimming over the 5 days during the learning curve of the MWM

Table S1: List of primary and secondary antibodies

| **Antibody** | **Reference** |
| --- | --- |
| Goat anti-CD31 | R&D #AF3628 |
| Rat anti-CD31 | Novus #NB600-1475 |
| Rat anti-CD34 | Novus #NB600-1071 |
| Rabbit anti-p21 | Abcam #ab188224 |
| Goat anti-Angptl2 | ThermoFisher #PA5-47139 |
| Rabbit anti-TNFα | GenTex #GTX110520 |
| Rabbit anti-Collagen IV | Abcam #ab19808 |
| Rat anti-IBA1 | Gentex #GTX635400 |
| Rabbit anti-Synaptophysin | #SAB4502906 |
| Chicken anti-GFAP | GenTex #GTX85454 |
| DAPI | Thermo Fisher #D1306 |
| Donkey anti-goat 555 | Thermo Fisher #A21432 |
| Donkey anti-rat 488 | Thermo Fisher #A21208 |
| Goat anti-chicken 488 | Thermo Fisher #A11039 |
| Goat anti-rabbit 647 | Thermo Fisher #A31573 |

Table S2: List of primers used for real-time quantitative PCR

| **Target gene** | **Primers** | **Sequences** |
| --- | --- | --- |
| *Angptl2* | Forward | GATCCAGAGTGACCAGAATC |
|  | Reverse | TCTCAGGCTTCACCAGGTAG |
| *PAI1* | Forward | TTGTCCAGCGGGACCTAGAG |
|  | Reverse | AAGTCCACCTGTTTCACCATAGTCT |
| *CD34* | Forward | TGAGATGACATCACCCACCG |
|  | Reverse | GCCAACCTCACTTCTCGGAT |
| *CD31* | Forward | CAACAGAGCCAGCAGTATGAG |
|  | Reverse | ATGACAACCACCGCAATGAG |
| *Tnfα* | Forward | CAAGTGGAGGAGCAGCTGGA |
|  | Reverse | CATCGGCTGGCACCACTAGT |
| *Il6* | Forward | GGATACCACTCCCAACAGACC |
|  | Reverse | TTCTGCAAGTGCATCATCGT |
| *Icam-1* | Forward | CAATTCACACTGAATGCCAGCTC |
|  | Reverse | CAAGCAGTCCGTCTCGTCCA |
| *Gfap* | Forward | CGAGATCGCCACCTACAGGA |
|  | Reverse | CGGATCTGGAGGTTGGAGAA |
| *p21* | Forward | TGTCGCTGTCTTGCACTCT |
|  | Reverse | AGACCAATCTGCGCTTGGA |
| *Ocln* | Forward | GCGGAAAGAGTTGACAGTCC |
|  | Reverse | CTTCAGGCACCAGAGGTGTT |
| *Cldn5* | Forward | TCGGGTGAGCATTCAGTCTT |
|  | Reverse | GTCACGATGTTGTGGTCCAG |
| *Bdnf* | Forward | AAGCCGCAAAGAAGTTCCAC |
|  | Reverse | CTTGTCCGTGGACGTTTACT |
| *NeuN* | Forward | CACAGCCCATTGCTGGGA |
|  | Reverse | TGAACCGGAAGGGGATGTTG |
| *Syp* | Forward | AGGGTGGTTATCAACCCGAT |
|  | Reverse | TGGGCTTCACTGACCAGATT |
| *CycloA* | Forward | CCGATGACGAGCCCTTGG |
|  | Reverse | GCCGCCAGTGCCATTATG |
| *HPRT* | Forward | GGTTAAGCAGTACAGCCCCA |
|  | Reverse | GGCCTGTATCCAACACTTCG |
| *BM2* | Forward | TCTCACTGACCGGCCTGTAT |
|  | Reverse | GATTTCAATGTGAGGCGGGTG |
| *PPIA* | Forward | CCACCGTGTTCTTCGACATC |
|  | Reverse | CTGGCACATGAATCCTGGAA |
|  | | |

Table S3: Impact of preventive (from 3-6m) and curative (from 9-12m) senolytic treatment on blood markers:

| **Preventive**  **3-6m** | **Males-**  **Placebo** | **Males-**  **ABT-263** | **Females-Placebo** | **Females-**  **ABT-263** |
| --- | --- | --- | --- | --- |
| Glucose (mmol/L) | 15.9±0.4 (11) | 17.2±0.5 (9) | 14.2±1.1 (8) | 15.6±0.7 (8) # |
| Cholesterol (mmol/L) | 23.2±1.0 (10) | 23.2±0.9 (9) | 25.0±1.0 (9) | 25.7±1.1 (8) |
| Triglycerides (mmol/L) | 6.5±0.4 (10) | 6.6±0.3 (9) | 4.5±0.2 (9) # | 4.4±0.5 (8) # |
| Urea (mmol/L) | 8.2±0.4 (10) | 8.2±0.5 (9) | 10.4±0.5 (9) # | 9.3±0.6 (8) |
| AST (U/L) | 250.0±91.3 (8) | 86.7±14.3 (9) | 198.3±61.4 (9) | 262.5±74.3 (6) |
| Phosphatase alkaline (U/L) | 65.5±3.5 (10) | 68.9±3.2 (9) | 112.2±4.6 (9) # | 98.8±3.8 (8) ***** # |

| **Curative**  **9-12m** | **Males-**  **Placebo** | **Males-**  **ABT-263** | **Females-Placebo** | **Females-**  **ABT-263** |
| --- | --- | --- | --- | --- |
| Glucose (mmol/L) | 13.1±1.7 (10) | 14.4±0.8 (12) | 16.6±1.3 (11) # | 14.5±0.9 (12) |
| Cholesterol (mmol/L) | 27.2±2.5 (10) | 29.4±1.6 (12) | 35.3±1.8 (11) # | 27.6±0.9 (12) ***** |
| Triglycerides (mmol/L) | 8.1±0.7 (10) | 7.2±0.3 (12) | 7.2±0.4 (11) # | 5.2±0.4 (12) ***** # |
| Urea (mmol/L) | 9.1±0.7 (10) | 9.4±0.7 (12) | 9.6±0.7 (11) | 9.6±0.7 (12) |
| AST (U/L) | 384.0±110.4 (10) | 248.5.7±48.9 (11) | 298.5±48.9 (9) | 480.0±134.6 (12) |
| Phosphatase alkaline (U/L) | 59.3±3.7 (7) | 66.7±6.0 (12) | 85.9±6.7 (11) # | 89.8±6.1 (12) # |

Data are mean±SEM of (n) mice; 2-way ANOVA, (Sex x Treatment) and Sidak’s multiple comparisons test. *****: p<0.05 vs Placebo (within the same sex). #: p<0.05 vs Males (within the same treatment).

**Suppl. Fig. 1**


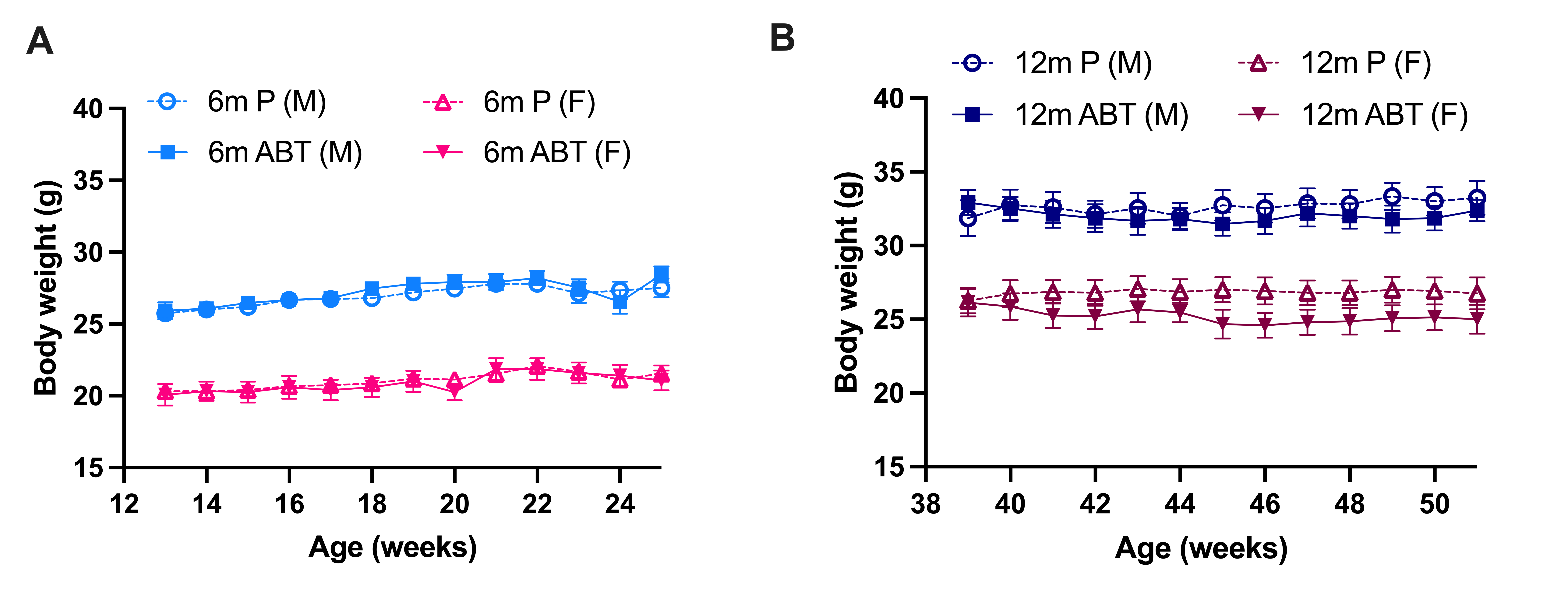


Mice body weight follow-up from (**A**) 3 to 6-month-old and (**B**) 9 to 12-month-old in male (M) and female (F) mice treated with placebo (P) or ABT-263 (ABT). Data are means±SEM of n=15 mice in each group.

**Suppl. Fig.2**





(**A**) **Y Maze**. Left, Percentage of alternations in the Y maze test in males (M) and females (F) ATX mice treated with placebo (P) or ABT-263 (ABT) at 6-month-old (6m) or 12month-old (12m). Right, Number of arm entries in the Y maze test in males and females ATX mice treated with placebo or ABT-263 at 6m or 12m. The minimal number of entries for the test being successful is 8 arm entries (illustrated by the dotted line). In young 6-mo mice, the Y maze test was unsuccessful in ~37% of the mice, of both sexes, treated or not (males-placebo: 6/18=33%; males-ABT: 7/19=37% failure; females-placebo: 8/19=42%; females-ABT: 7/20=35% failure). In older 12-mo mice, the test was unsuccessful in 100% of the mice. Data are mean±SEM of n=18-20 (6-mo) and n=11-13 (12-mo) mice. ANOVA-2way (Sex X Treatment) and Sidak’s multiple comparisons test.

(**B**) **NOR validation** Untreated male ATX mice of 6 months of age were subjected to NOR. The test comprises 3 trials (habituation in an open field; training with the exploration of 2 identical objects; test after the introduction of a novel object) performed within the same day. A computer-based video-tracking system recorded the movement (times [s] spent in exploring familiar *versus* novel object). The test was considered functional, since 1) the time spent on both objects was higher than 20 seconds; 2) during the training trial, the time spent on familiar objects was similar (Time spent on Object-1 *vs.* Object-2: 36.9±6.7 *vs* 42.4±11.6 s, n=23, p=0.708); 3) during the test trial, the time spent on the novel object was significantly higher (Time spent on Object-1 vs Novel Object-2: 18.5±4.0 *vs* 61.7±21.4 s, n=23, p=0.008), demonstrating that mice discriminate the novel from the familiar object. Data are mean±SEM of 23 mice. *: p<0.05 *vs.* familiar object-1, Wilcoxon matched-pairs signed rank test. (**C**) **Recognition index in the Novel object recognition** (NOR) test in both males (M) and females (F) mice treated with placebo (P) or ABT-263 (ABT) at 6-month-old (6m) or 12month-old (12m). (**D, E**) Total distance (cm) and velocity of the movement of mice during the NOR test, in placebo and ABT-263 treated mice. Preventive (from 3 to 6m) and curative (from 9 to 12m) treatments had no effect on mobility. Data are mean±SEM of n=11-15 mice in placebo and ABT-263-treated mice. ANOVA-2way (Sex X Treatment) and Sidak’s multiple comparisons test.

**Suppl. Fig.3**


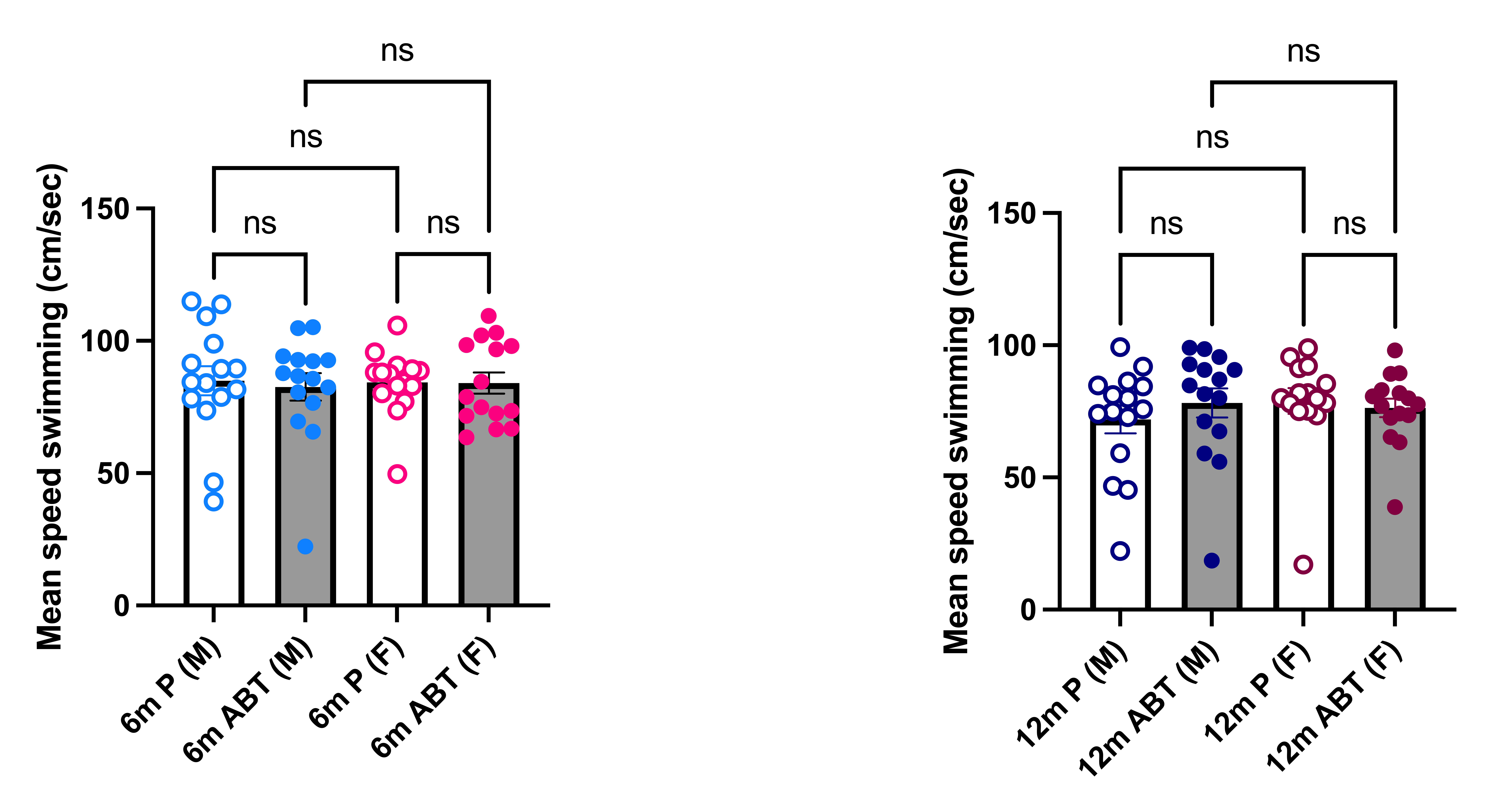


Total mean speed of swimming over the 5 days during the learning curve of the MWM. Data are mean±SEM of n=15 mice in placebo and ABT-263-treated mice. *: p<0.05, ANOVA-2way (Sex X Treatment) and Tukey’s multiple comparisons test.
